# Cecum microbiome and metabolism characteristics of Silky Fowl and White Leghorn chicken in late laying stages

**DOI:** 10.3389/fmicb.2022.984654

**Published:** 2022-10-20

**Authors:** Xue Yang, Yurong Tai, Yuhao Ma, Zihan Xu, Jiaqi Hao, Deping Han, Junying Li, Xuemei Deng

**Affiliations:** ^1^Key Laboratory of Animal Genetics, Breeding and Reproduction of the Ministry of Agriculture, College of Animal Science and Technology, China Agricultural University, Beijing, China; ^2^College of Veterinary Medicine, China Agricultural University, Beijing, China

**Keywords:** White Leghorn, Silky Fowl, cecal microbiome, cecal metabolome, correlation analysis

## Abstract

Cecal microflora plays a key role in the production performance and immune function of chickens. White Leghorn (WL) is a well-known commercial layer line chicken with high egg production rate. In contrast, Silky Fowl (SF), a Chinese native chicken variety, has a low egg production rate, but good immune performance. This study analyzed the composition of cecal microbiota, metabolism, and gene expression in intestinal tissue of these varieties and the correlations among them. Significant differences were observed in the cecal microbes: *Bacteroides* was significantly enriched in WL, whereas *Veillonellaceae* and *Parabacteroides* were significantly enriched in SF. Carbohydrate biosynthesis and metabolism pathways were significantly upregulated in WL cecum, which might provide more energy to the host, leading to persistently high levels of egg production. The higher *Parabacteroides* abundance in SF increased volicitin content, enhanced α-linolenic acid metabolism, and significantly negatively correlated with metabolites of propanoate metabolism and carbohydrate metabolism. Genes related to lipid metabolism, immunity, and melanogenesis were significantly upregulated in the SF cecum, regulating lipid metabolism, and participating in the immune response, while genes related to glucose metabolism and bile acid metabolism were expressed at higher levels in WL, benefiting energy support. This study provided a mechanism for intestinal microorganisms and metabolic pathways to regulate chicken egg-laying performance and immunity.

## Introduction

Gut microbes have been shown to play a key role in physiological activities, such as obesity (Maruvada et al., 2017), immune coordination (Zheng et al., 2020), metabolism (Postler and Ghosh, 2017), and host gene expression regulation (Collins and Patterson, 2020). Intestinal microorganisms directly stimulate or indirectly (metabolites) affect the host’s intestinal function, which in turn affects the host’s absorption and utilization of dietary nutrients and regulates production performance and immune function (Hill and Round, 2021). The small intestine is the main site of nutrient digestion and absorption. The cecum is a major part of the large intestine, where microbial fermentation produces volatile fatty acids, which provide energy for the host (Binek et al., 2017). Fermentation of cecal microbiota contributes to the health and productivity of chickens and helps resist colonization by invading pathogens (Bjerrum et al., 2006; Saxena et al., 2016).

The gut microbiota is closely related to poultry productivity (Diaz Carrasco et al., 2019). Chen et al. (2019) showed that the cecal microbiome and metabolites contribute more efficiently to a higher growth performance in chickens. Yan et al. (2017) showed that *Lactobacillus* and *Akkermansia* had significantly higher abundance in the cecal contents of hens with better feed efficiency and enriched functions related to carbohydrate and amino acid metabolism, suggesting that *Lactobacillus* can improve the feed efficiency of hosts. In addition to determining the role of the gut microbiome in growth (Rubio et al., 2015), studies have found that probiotic supplementation can improve laying performance, egg quality, and hatching in laying hens (Mikulski et al., 2020). Therefore, intestinal microbes may be potential targets for regulating chicken production performance.

Regarding the effects of intestinal microbes on immune regulation, preliminary studies on germ-free chickens suggest that microbial exposure is necessary for proper development and maturation of the intestinal immune system (Dibner et al., 1998). Yang et al. (2014) showed that *Lactobacillus* strains could significantly reduce the expression of *Salmonella* virulence genes and protect body health. Probiotics supplementation can change intestinal flora, stimulate the immune system, reduce inflammation, and prevent colonization by pathogenic bacteria (Jha et al., 2020).

Microbial colonization may be host dependent and species specific. Waite and Taylor showed that although sampling location, diet, and captivity status play a role, the host is the most important factor determining microbial community composition (Waite and Taylor, 2014). Studies have shown the presence of *Bacteroidetes* in the ileal contents of 20-day-old Cobb broilers but not in Ross broilers. However, *Actinomycetes* was present in the ileal contents of Ross broilers, but not in Cobb broilers (Nakphaichit et al., 2011; Kim J. E. et al., 2015). When maintaining the same growth environment, there are differences in the composition of intestinal microbes among different breeds or strains of chickens. Pandit et al. (2018) found significant differences in the cecal microbial community structure between Ross and Cobb broilers and Indian native breeds of the same age. Bidirectional selection for specific economic traits also has significant effects on intestinal flora (Schokker et al., 2015). Yang et al. (2017) observed significant differences in fecal flora composition between strains using chickens from the 40th generation of a bidirectional antibody titer breeding line at Virginia Tech. Some researchers have also observed microbial differences in the feces of high-fat and low-fat hens (Ding et al., 2016). Kundu et al. (2016) evaluated the immune competence of native and White Leghorn (WL). Native chickens showed a higher haemagglutinin test, while WL showed the lowest response and highest mortality.

In this study, we compared the cecal microbiome, metabolome, and tissue transcriptome between WL and Silky Fowl (SF) chickens. WL is a well-known commercial layer line with early maturity, high egg productivity, and low feed consumption (Allonby and Wilson, 2018). SF is a local breed with lower egg productivity and strong immunity, with widely distributed melanocytes. Melanocytes play an important role in innate immunity during viral infection (Han et al., 2021). Chickens were raised in the same chicken house, fed the same feed, and had similar growth curves. We investigated the effects of cecal microorganisms and host genes on egg-laying and immune differences between the two varieties. This may provide a way to improve egg productivity or immunity through transplantation of dominant flora.

## Materials and methods

### Sample collection and index determination

WL and SF hens of the same age in separate conservation populations were raised in the Experimental Unit for Poultry Genetic Resource and Breeding, with the same feeding, management, and environmental conditions; single cage feeding; and free access to feed and water. Laboratory Animal Welfare Experiment License from China Agricultural University was obtained (permit number: SKLAB-2012-04-07). All experiments were approved by the Committee on the Animal Experimental Ethical Inspection of China Agricultural University (issue number: AW32802202-1-1).

Eight SF and eight WL hens (48-weeks old) were sacrificed by severing the jugular veins after anesthesia after weighing, bled for 3–5 min, and then dissected. The luminal contents of cecum samples were collected after slaughter. Liver weight and cecum length were determined, and the liver index was calculated as: liver index = (liver weight/body weight) × 100%.

Cecum tissue were cut into two along the sagittal plane; one half was fixed in 4% paraformaldehyde solution (Beijing Solarbio Life Science and Technology Co., Ltd., Beijing, China), and the other half was stored in liquid nitrogen and then transferred into –80°C freezer until use.

### Oil red O staining

After fixation in 4% paraformaldehyde solution for a minimum period of 24 h before use, the caeca were trimmed and dehydrated by 30 and 50% sucrose solution, and then embedded in OCT (Opti-mum Cutting Temperature compound, Leica, Shanghai, China) to prepare 15 mm frozen sections. Sections were washed with distilled water and incubated in oil red O for 10 min. After being rinsed with isopropanol for 2 s and distilled water for 1 s, the sections were rinsed with Hematoxylin solution for 5 min, and then incubated in distilled water for 10 min. And then the sections were mounted with neutral balsam for observation under light microscope. The histopathological changes were observed and pictured using a Zeiss camera system (Carl Zeiss Optics Co., Ltd., Guangzhou, China).

### 16S rRNA gene amplicon sequencing

Total genomic DNA samples of cecum contents were extracted using the OMEGA Soil DNA Kit (D5625-01) (Omega Bio-Tek, Norcross, GA, USA), following the manufacturer’s instructions, and stored at –20°C prior to further analysis. PCR amplification of the bacterial 16S rRNA genes V4-V5 region was performed using the forward primer 515F (5′- GTGCCAGCMGCCGCGGTAA-3′) and the reverse primer 907R (5′- CCGTCAATTCMTTTRAGTTT-3′). After the individual quantification step, amplicons were pooled in equal amounts, and pair-end 2,250 bp sequencing was performed using the Illumina MiSeq platform with MiSeq Reagent Kit v3 at Shanghai Personal Biotechnology Co., Ltd. (Shanghai, China).

Microbiome bioinformatics were performed with QIIME2 2019.4 (Bolyen et al., 2019) with slight modification according to the official tutorials.^1^ Briefly, raw sequence data were demultiplexed using the demux plugin, followed by primer cutting with cutadapt plugin (Martin, 2011). Sequences were then quality filtered, denoised, merged, and chimera removed using the DADA2 plugin (Callahan et al., 2016). Non-singleton amplicon sequence variants (ASVs) were aligned with mafft (Katoh et al., 2002) and used to construct a phylogeny using fasttree2 (Price et al., 2010). Sequence data analyses were mainly performed using QIIME2 (Caporaso, 2019) and R packages (v3.2.0). ASV-level alpha diversity indices, such as Chao1 richness estimator, Observed species, Shannon diversity index, Simpson index, Faith’s PD, Pielou’s evenness and Good’s coverage were calculated using the ASV table in QIIME2, and visualized as box plots. The number under the label of diversity index is the *P*-value tested by Kruskal-Wallis test.

Beta diversity analysis was performed to investigate the structural variation of microbial communities across samples using Bray-Curtis metrics and visualized *via* principal coordinate analysis (PCoA). The taxonomy compositions and abundances were visualized using MEGAN and GraPhlAn. Linear discriminant analysis (LDA) effect size (LEfSe) method^2^ was used to perform cecal microbiota features differentiating. LEfSe uses the Kruskal-Wallis rank sum test to detect features with significantly different abundances between assigned taxa and an effect size threshold of 3.5 were used for all biomarkers discussed in this study. Microbial functions were predicted by PICRUSt2 (Phylogenetic investigation of communities by reconstruction of unobserved states) using ASVs sequence and abundance, upon MetaCyc Metabolic Pathway Database (MetaCyc),^3^ Kyoto Encyclopedia of Genes and Genomes Database (KEGG)^4^ and Cluster of Orthologous Groups of proteins Database (COG)^5^ databases. All sequences were deposited in the National Center for Biotechnology Information (NCBI) and can be accessed in the Short Read Archive (SRA) under the accession number PRJNA848375.

### Untargeted metabolomics

In this experiment, HILIC UHPLC-Q-EXACTIVE MS technology combined with data-dependent acquisition method was used to analyze the full spectrum of the sample, and the primary and secondary mass spectrometry data were obtained at the same time, and then Compound Discoverer 3.0 (Thermo Fisher Scientific) was used to perform peak extraction and metabolite identification of the data.

To monitor the stability and repeatability of instrument analysis, quality control (QC) samples were prepared by pooling 10 μl of each sample and analyzed together with the other samples. The ACQUITY UPLC BEH C18 column (100 mm*2.1 mm, 1.7 μm, Waters, USA) was used for chromatographic separation. The mobile phase A was water and 0.1% formic acid, B mobile phase is acetonitrile. The loading volume for each sample is 5 μl. The sample was placed in the 4°C autosampler during the entire analysis. In order to avoid the influence caused by the fluctuation of the detection signal of the instrument, a random order is adopted for continuous analysis of samples. QC samples are inserted after each group of samples in the sample queue to monitor and evaluate the stability of the system and the reliability of experimental data.

Electrospray ionization (ESI) positive ion and negative ion modes were used for detection. The samples were separated by UHPLC and analyzed by Q-Exactive quadrupole-electrostatic field orbitrap high-resolution mass spectrometer (Thermo Fisher Scientific). In the extracted ion features, only the variables having more than 50% of the non-zero measurement values in at least one group were kept. Compound identification of metabolites was performed by comparing of accuracy m/z value (0<25 ppm), and MS/MS spectra with an in-house database established with available authentic standards. After normalized to total peak intensity, the processed data were uploaded, then imported into SIMCA-P (version 14.1, Umetrics, Umea, Sweden), where they were subjected to multivariate data analysis, including Pareto-scaled principal component analysis (PCA) and orthogonal partial least-squares discriminant analysis (OPLS-DA). The variable importance in the projection (VIP) value of each variable in the OPLS-DA model was calculated to indicate its contribution to the classification. Metabolites with the VIP value > 1 was further applied to Student’ s *t*-test at univariate level to measure the significance of each metabolite, the *p*-values less than 0.05 were considered as statistically significant. The significant difference metabolites were screened, and then cluster analysis and KEGG metabolic pathway analysis were performed on the difference metabolites.

### Transcriptome sequencing

Cecum tissues were collected and the total RNA was extracted by using TRIZOL reagent (Invitrogen, USA) according to the manufacturer’s protocol. RNA purity and quantification were evaluated using the NanoDrop 2000 spectrophotometer (Thermo Fisher Scientific, USA). RNA integrity was assessed using the Agilent 2100 Bioanalyzer (Agilent Technologies, Santa Clara, CA, USA). Libraries were constructed using the TruSeq™ RNA Sample Prep kit (Illumina, San Diego, CA, USA) according to the manufacturer’s instructions. Sequencing of the libraries was performed on an Illumina HiSeq2000 instrument by Shanghai Personal Biotechnology Co., Ltd. (Shanghai, China). The sequencing data contained a few connectors and low-quality Reads, and thus Cutadapt (v1.15) software was used to filter the sequencing data to get high-quality sequence (Clean Data) for further analysis. Reads with number of expected errors higher than [default: 2.0] were discarded. The clean reads were mapped to the chicken genome (GRCg6a/galGal6) using HISAT2. FPKM of each gene was calculated using Cufflinks, and the read counts of each gene were obtained by HTSeq-count. Differential expression analysis was performed using the DESeq (2012) R package. Padjust value < 0.05 [Benjamini-Hochberg (BH) multiple test correction], and fold change > 2 was set as the threshold for significant differential expression. Hierarchical cluster analysis of differentially expressed genes (DEGs) was performed to demonstrate the expression pattern of genes in different groups and samples. Top GO (2.40.0) was used for Gene Ontology (GO) enrichment analysis of the DEGs, and ClusterProfiler (3.16.1) software was used for KEGG pathway enrichment analysis to understand the high-level functions and utilities of the biological system. All RNA sequences were deposited in the NCBI and can be accessed in the SRA under the accession number PRJNA848673.

### Statistical analysis

All statistical analyses were performed using Prism 6.0 (GraphPad Software, San Diego, CA). Data were expressed as means ± standard error (M ± SE). Statistical significance was evaluated using Student’s *t*-test. The redundancy analysis (RDA) was performed by the genescloud tools, a free online platform for data analysis.^6^ MetOrigin^7^ was used to integrate the statistical correlations and biological relationships between microbiome and metabolomics (Yu et al., 2022). Statistically, Spearman correlation analysis was chosen in MetOrigin. Biologically, each metabolite was searched against the KEGG database to identify bacterial species that could participate in a metabolic reaction. Sankey network diagrams were used to integrate and demonstrate statistically and biologically significant associations between microorganisms and metabolites. Differential metabolites from host, microbiota, and co-metabolic sources, and related bacteria were integrated, respectively, to obtain microbial and metabolite interaction networks.

## Results

### Phenotypic characteristics of White Leghorn and Silky Fowl

**FIGURE 1:**
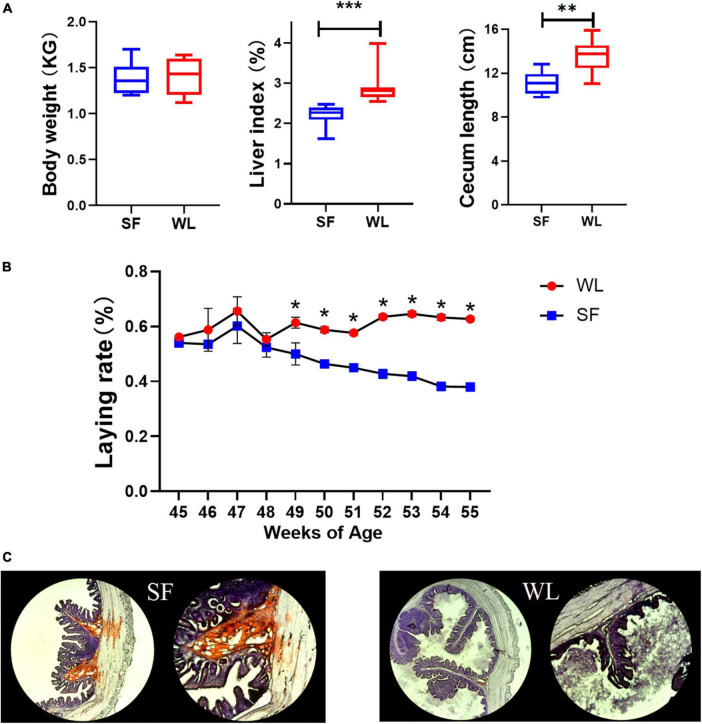
Phenotypic index and oil red O staining. **(A)** Comparison of WL and SF in weight, liver index and cecum length. **(B)** Laying rate of SF and WL flock from 45 to 55-week-old. **(C)** Histological observation of WL and SF cecum after oil red O staining. Asterisk coding is indicated in the *P < 0.05; **P < 0.01; ***P < 0.001.

There was no significant difference in body weight between SF and WL, but the liver index and cecum length of SF were significantly lower than those of WL (Figure 1A). After 45 weeks of age, the SF egg-laying rate showed an obvious downward trend, while that of WL remained relatively stable at a high level (Figure 1B). Oil Red O staining showed that positive sites were mainly located in the submucosa of the cecum, and the lipid content of SF was significantly higher than that of WL (Figure 1C).

SF and WL had similar body size, but SF deposited more fat in the intestines and WL produced more eggs, that is, formed more yolks, which may indicate different mechanisms of fat formation and distribution between the two breeds.

### Differential microbiome composition and potentially functional prediction in the cecum of White Leghorn and Silky Fowl

A total of 1,512,400 raw reads (2 × 250 bp) were obtained by 16S rRNA gene sequencing, and 1,352,777 reads passed the filtering, with an average value of 84,548 reads/sample (SD: 15,686) and a median sequence length of 441 bp. To avoid potential biases due to different sequencing depths, all samples were rarefied at 3,000 reads after raw read quality filtering. Rarefaction analysis and Good’s coverage indicated satisfactory coverage for all samples (average Good’s coverage of 97.76%).

**FIGURE 2:**
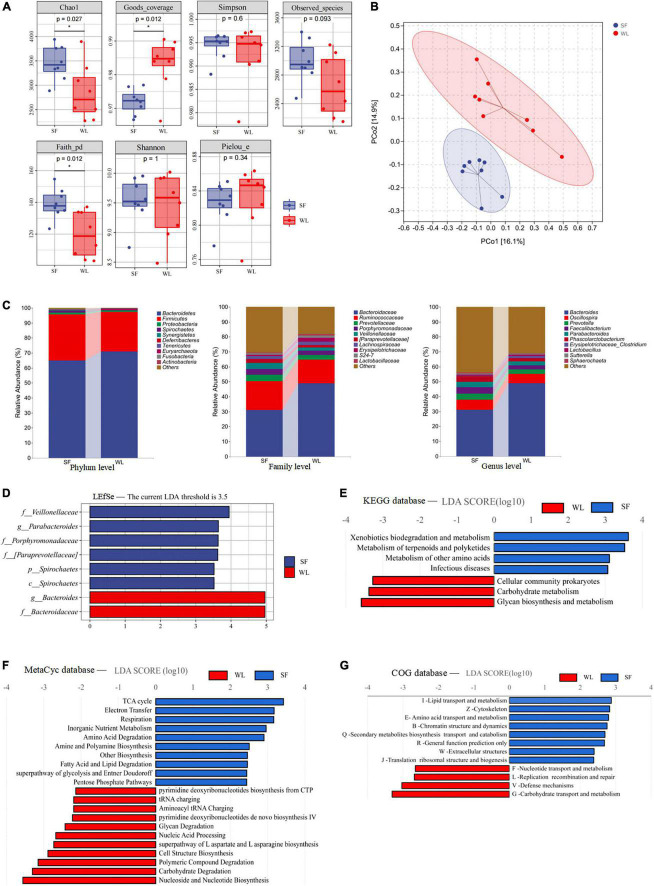
Cecal microbiome description of WL and SF. **(A)** Cecal Microbial Alpha Diversity Index Diagram of WL and SF. **(B)** Principal coordinate analysis (PCoA) based on Bray-Curtis distance, with 95% ellipse confidence. **(C)** Average relative abundances of dominant bacterial phylum, family and genus. **(D)** LEfSe analysis with LDA threshold 3.5, and metabolism pathways prediction of the cecal microbiota based on KEGG **(E)**, MetaCyc **(F)**, and COG **(G)** databases. Statistical significance is given as ***P < 0.001; **P < 0.01; *P < 0.05. Brackets are used to emphasize that this is the official Greengenes database recommended taxonomic information, there may be corrections or improvements to this taxonomic information.

Compared to WL, SF had higher Chao1 and Faith pd indices but lower Good’s coverage index (Figure 2A). The results showed that microbial community richness of the SF cecum was higher than that of WL chickens (*P* < 0.05), and the proportion of unclassified microbial species in SF cecal microbe samples was higher (*P* < 0.05). β-diversity analysis was used to compare the overall microbial profiles of all the groups as displayed in Figure 2B. PCoA was performed to present a holistic perception of the microbiota. The results of PCoA showed that the groups were mainly organized into two clusters, which illustrated that the microbiota composition in each group was dissimilar. The samples from each group were fully aggregated; moreover, the intestinal microbial flora between the SF individuals was more uniform and similar.

Sequence alignment and annotation showed that most microbiota taxa belonged to 15 phyla, among which the dominant phyla were Bacteroides (67.97%) and Firmicutes (28.61%). The high proportion of Bacteroides in both groups indicated that this phylum was consistent as the dominant phylum, especially in WL (71.05%), where Bacteroides exhibited a higher dominance than that in SF (64.89%). Although Bacteroides was the dominant phylum in SF, the abundances of Firmicutes and Spirochetes (*P* < 0.05) in SF were higher than those in WL (Figure 2C). At the family level, the relative abundance of *Bacteroidaceae* was significantly lower in SF (31.18%) than in WL (49.00%), but the abundances of *Porphyromonadaceae*, *Veillonellaceae* (*P* < 0.05), and *[paraprevotellaceae]* (*P* < 0.05) were higher in SF than in WL. At the genus level, these microbes belonged to > 150 genera. Differences were also observed at the genus level: *Bacteroides* was significantly higher in WL (48.89%) than in SF (31.05%), while *Faecalibacterium*, *Parabacteroides*, and *Phascolarctobacterium* (*P* < 0.05) were higher in SF.

LEfSe was used to analyze the differential abundances of bacterial taxa. Specifically, *Bacteroides* and *Bacteroidaceae* were enriched in WL, *Veillonellaceae*, *Parabacteroides*, *Prophyromonadaceae*, *[Paraprevotellaceae]*, and Spirochetes were enriched in SF (Figure 2D). In the KEGG (Figure 2E) database, the enriched pathways of WL were glycan biosynthesis and metabolism, and carbohydrate metabolism, while those of SF were xenobiotics biodegradation and metabolism. Using the MetaCyc (Figure 2F) database, the enriched pathways of WL were carbohydrate degradation and glycan degradation, while in SF, the enriched pathways were the TCA cycle and amino acid, fatty acid and lipid degradation. In the COG (Figure 2G) database, the enriched pathways of WL were carbohydrate transport and metabolism, while those in SF were lipid transport and metabolism as well as amino acid transport and metabolism.

### Metabolomic difference of cecal contents in White Leghorn and Silky Fowl

**FIGURE 3:**
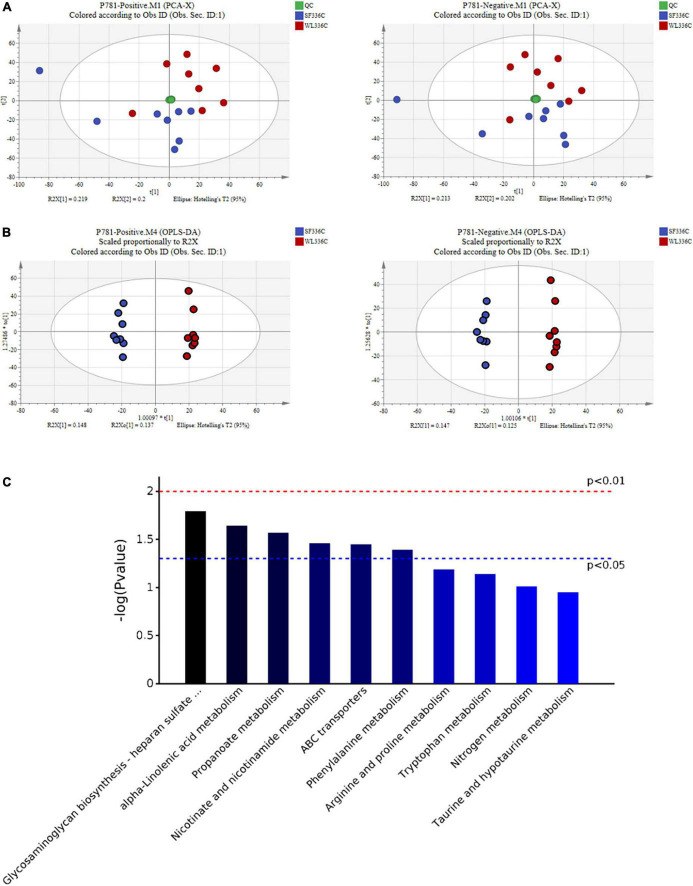
The pattern discriminant and composition description of cecal metabolites in WL and SF. **(A)** PCA pattern recognition in positive ion (ESI +) and negative ion (ESI-) mode. **(B)** Discrimination of OPLS-DA mode in positive ion (ESI +) and negative ion (ESI-) mode. **(C)** The Bar chart of differential metabolic pathways.

To characterize the metabolite changes induced in cecal contents, we performed LC-MS/MS-based metabolomic analysis of WL and SF. PCA analysis showed that all QC samples were close and well gathered near the ordinate origin, indicating that the detection platform was stable, and the instrument precision was good. SF and WL were clearly distinguished, indicating that there were notable differences in cecal metabolites (Figure 3A). In Hotelling T2 Ellipse, the cumulative values of R2Y and Q2 were both greater than 0.6, indicating that the OPLS-DA model could explain the difference between the two groups of samples well, and further confirming that there was a significant difference between SF and WL cecal content-related metabolic components (Figure 3B). The OPLS-DA model R2Y was very close to 1, which showed that the established model conformed to the real situation of the sample data (Supplementary Figures 1A,B).

The differential metabolites were screened using a *t*-test where *P* < 0.05 indicated a significant difference (Supplementary Figures 1C,D). In total, 271 potential chicken cecal metabolic markers that could distinguish the SF and WL groups were screened. SF significantly upregulated 103 metabolites and downregulated 168 differential metabolites, which may have important biological functions in the cecum of SF and WL (Supplementary Table 1).

We submitted the differential metabolites (including positive and negative ion model results) obtained in the sample group to the KEGG website for relevant pathway analysis. The most significantly different metabolic pathways were α-linolenic acid metabolism, nicotinate, and nicotinamide metabolism, phenylalanine metabolism, glycosaminoglycan biosynthesis-heparan sulfate/heparin, propanoate metabolism, and ABC transporters (Figure 3C and Table 1).

**TABLE 1:** Differential metabolites that mapped to KEGG pathways.

Metabolites name	Metabolites ID	Log2FoldChange	*P*-value (SF/WL)	VIP	KEGG pathway
Acetate	POSM0714	–0.6406	0.04554	1.3226	Glycosaminoglycan biosynthesis—heparan sulfate/heparin Propanoate metabolism
Methyl jasmonate	NEGM0467	–0.4540	0.04338	1.3943	Alpha-Linolenic acid metabolism
Volicitin	NEGM0675 POSM0009	3.6490	0.00149 0.00145	1.8561 1.8450	Alpha-Linolenic acid metabolism
Propanoate	NEGM0440 POSM0593	–0.9790	0.00453 0.00437	1.6511 1.6651	Propanoate metabolism Nicotinate and nicotinamide metabolism
6-Hydroxypseudooxynicotine	NEGM0277	0.7713	0.03439	1.4373	Nicotinate and nicotinamide metabolism
Deoxyuridine	NEGM0253 POSM0421	–1.0769	0.04252 0.04696	1.4041 1.3637	ABC transporters
L-Proline	POSM0267	–0.8134	0.035234	1.3343	ABC transporters
Spermidine	NEGM0007	–0.6982	0.01387	1.5735	ABC transporters
Phenylpropanoate	NEGM0692 POSM0580	–1.3395	0.03302 0.03331	1.3664 1.3591	Phenylalanine metabolism
D-Cathinone	NEGM0408	0.8979	0.03081	1.4791	Phenylalanine metabolism

Acetate is produced in the modification stage of heparan sulfate biosynthesis, and its content in WL cecal content was 1.56 times that in SF, providing a substrate for WL energy metabolism. Propanoate, as an important product of propanoate metabolism and nicotinate and nicotinamide metabolism, that could provide ample energy to the body and basic substances for acetic acid metabolism, was significantly enriched in WL. Deoxyuridine, L-Proline, and spermidine are ABC transporters that are mainly responsible for transporting minerals, organic ions, and phosphate. Their concentrations increased significantly in WL, indicating that ABC transporters transported more ions and phosphates in WL, ensuring nutrition supply.

Volicitin is a fatty acid-amino acid conjugate obtained after the decomposition of α-linolenic acid. The content of volicitin in SF was 12.54 times that in WL, indicating that fatty acid was decomposed more in SF, while methyl jasmonate, one of the final products of α-linolenic acid metabolism, was relatively low in SF, indicating that fatty acids might conjugatively accumulate during metabolism. 6-Hydroxypseudooxynicotine, a compound of nicotine, in SF was 1.71 times that in WL and played a significant role in nicotinate and nicotinamide metabolism.

### Differentially expressed genes in White Leghorn and Silky Fowl hosts

**FIGURE 4:**
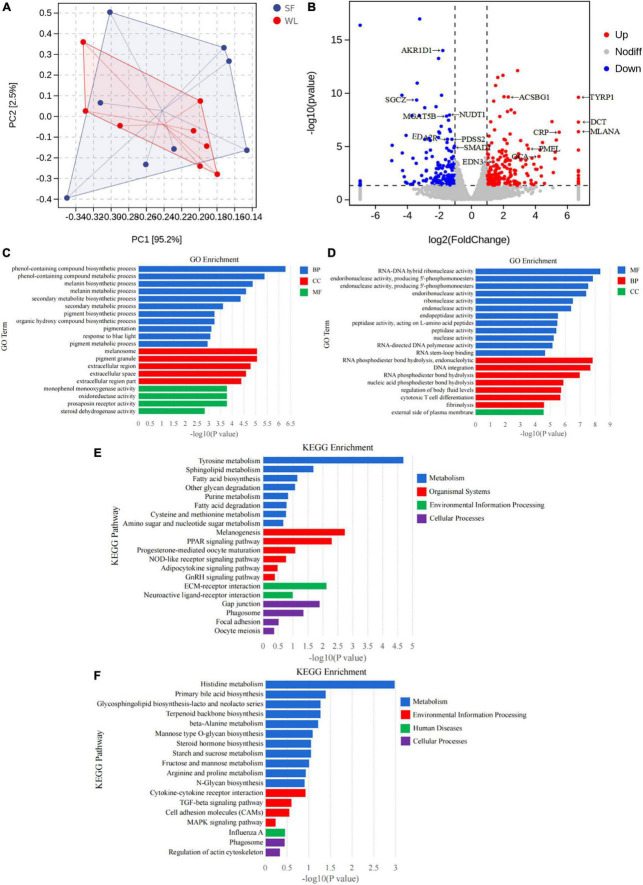
Differential characterization of cecum gene expression in hosts. **(A)** PCA map of cecal intestinal wall transcriptome. **(B)** Differential expression volcano plot in the cecal transcriptome of SF and WL. **(C)** GO classification of the up-regulated expressed genes of SF. **(D)** GO classification of the up-regulated expressed genes of WL. **(E)** KEGG classification of the up-regulated expressed genes of SF. **(F)** KEGG classification of the up-regulated expressed genes of WL.

There was a clear difference in the cecum gene expression profile between WL and SF, as revealed by the PCA plot (Figure 4A) and volcano plot (Figure 4B). A total of 397 DGEs were identified in the cecum tissue between the groups, including 232 upregulated and 165 downregulated genes in the SF group relative to those in the WL group (Supplementary Table 2).

In SF, GO clustering analysis showed that the upregulated DEGs were related to melanin biosynthetic process, melanin metabolic process, and pigment biosynthetic process (Figure 4C). In WL, the upregulated genes were related to RNA-DNA hybrid ribonuclease, endoribonuclease, endonuclease, and peptidase activity (Figure 4D).

The upregulated genes in the SF group were found to be involved in tyrosine metabolism, sphingolipid metabolism, fatty acid biosynthesis, fatty acid degradation, melanogenesis, and PPAR signaling pathway (Figure 4E); whereas those in the WL were involved in histidine metabolism, glycosphingolipid biosynthesis-lacto, and neolacto series, primary bile acid biosynthesis, mannose type O-glycan biosynthesis, starch and sucrose metabolism, fructose and mannose metabolism, and N-glycan biosynthesis (Figure 4F).

### Redundancy analysis and biological relationships integration

**FIGURE 5:**
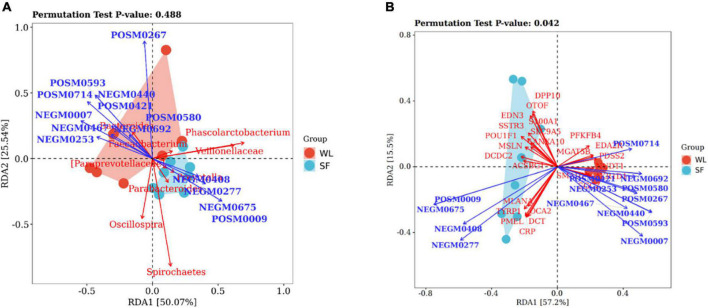
Redundancy analyses between microbes, metabolites and DEGs. **(A)** RDA between significantly different microbe and metabolite. **(B)** Relationship between metabolite and DEGs by RDA. Brackets are used to emphasize that this is the official Greengenes database recommended taxonomic information, there may be corrections or improvements to this taxonomic information.

RDA was used to determine the relationship between cecal microbiota and metabolites. It showed that *Bacteroides* in WL was positively correlated with acetate (*P* < 0.05), propanoate (*P* < 0.05), and spermidine (*P* < 0.05) (Figure 5A and Supplementary Table 3); while in SF, *Parabacteroides*, and *Veillonellaceae* were positively correlated with volicitin (*P* < 0.05), 6-hydroxypseudooxynicotine, and D-cathinone. These results indicated that *Bacteroidetes* in the WL cecum affected metabolite production of glycosaminoglycan biosynthesis, propanoate metabolism, and ABC transporter pathways, promoting energy generation and ion transport. Bacteria in the SF cecum increased the concentrations of metabolites from α-linolenic acid metabolism, phenylalanine metabolism, and nicotinate and nicotinamide metabolism, which resulted in fatty acid accumulation.

By exploring the correlation between metabolites and DEGs using RDA (Figure 5B and Supplementary Table 4), we found that genes related to melanin production (*MLANA*, *TYRP1*, *PMEL*, *DCT*, and *OCA2*) in SF were positively correlated with *CRP*, which was positively related to 6-hydroxypseudooxynicotine (*P* < 0.05) and D-cathinone (*P* < 0.05). The upregulation of melanin production genes in SF was in accordance with the upregulation of *CRP*. Volicitin (*P* < 0.05) was positively correlated with *ACSBG1*, and increased concentration of the fatty acid metabolite volicitin resulting in a significant upregulation of *ACSBG1* in the PPAR signaling pathway, which improved the level of fatty acid metabolism and immune response.

In WL, *PFKFB4*, *MGAT5B*, *EDA2R*, and *PDSS2* were positively correlated with acetate (*P* < 0.05); *SMAD1*, *NUDT1*, *AKR1D1*, and *SGCZ* were positively correlated with phenylpropanoate, deoxyuridine, L-proline, methyl jasmonate, propanoate (*P* < 0.05), and spermidine (*P* < 0.05). The increase in acetate concentration in WL cecum may cause the upregulation of glucose metabolism-related genes *PFKFB4* and *MGAT5B*, and the improvement in energy metabolism and ion transport could promote the upregulation of egg production-related genes *AKR1D1* and *EDA2R*.

**FIGURE 6:**
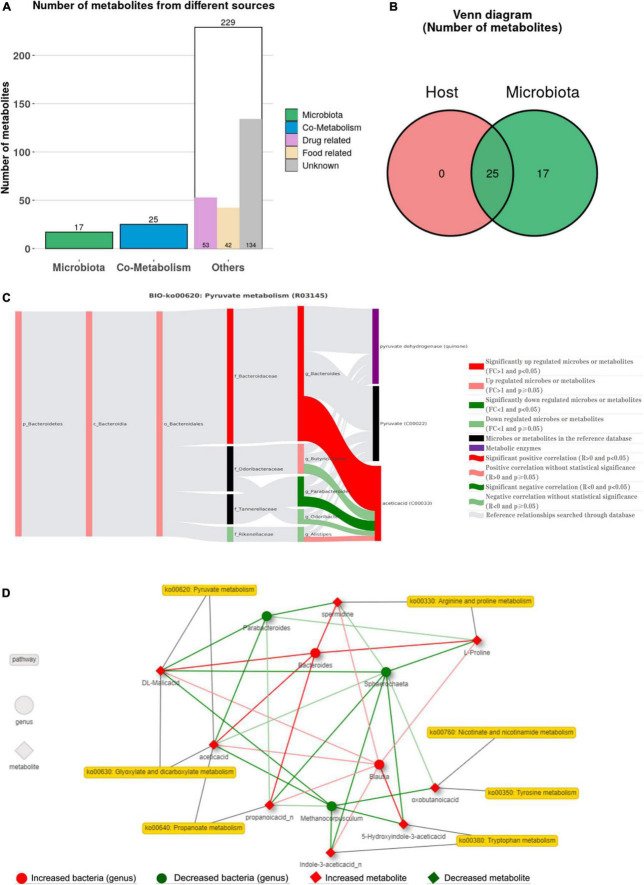
Correlation analyses between microbes and metabolites. **(A)** Venn diagram of the number of metabolic pathways in microbial community. **(B)** Venn diagram of the number of metabolites in bacterial communities. **(C)** The BIO-Sankey Network for R03145 metabolic reaction in pyruvate metabolism. **(D)** Network summary of co-metabolism pathways between metabolites and microbes.

The sources of 271 metabolites with significant differences were obtained using MetOrigin analysis (Figures 6A,B), of which 17 were microbial metabolites, 25 were co-metabolites of microbe and hosts, and 229 were other metabolites (53 drug-related, 42 food-related, and 134 unknown). Simultaneously, the metabolites with significant upregulation difference in WL and SF were also traced, the proportion of drug-related metabolites was larger in SF (29.13%) than in WL (13.69%) (Supplementary Figure 2). The obtained metabolites were compared with the KEGG database, and the metabolic pathways of bacterial metabolism and co-metabolism were determined (Supplementary Figure 3). Tryptophan metabolism, arginine and proline metabolism, nicotinate, and nicotinamide metabolism, tyrosine metabolism, and pyruvate metabolism were significantly different co-metabolism pathways. At the genus level, the Spearman correlation analysis between bacteria and metabolites is shown in Supplementary Figure 4, in which *Bacteroides* was significantly positively correlated with propanoic acid, while *Parabacteroides* was significantly positively correlated with volicitin.

Using metabolite traceability to determine bacterial flora participating in metabolic reactions, *Bacteroides* participated in pyruvate metabolism, propanoate metabolism, arginine and proline metabolism, and glycolysis/gluconeogenesis pathways, which significantly positively promoted the metabolites acetic acid, propanoic acid, L-proline, and spermidine, while *Parabacteroides* was significantly negatively correlated with the metabolites acetic acid and propanoic acid in pyruvate metabolism, propanoate metabolism, and glycolysis/gluconeogenesis pathways (Figure 6C and Supplementary Figure 5). Finally, more specific information on the metabolic changes of genus-level bacteria and metabolites was displayed through microbial-metabolite association network (Figure 6D). The co-metabolism network of the seven metabolic pathways showed that eight metabolites were associated with five differential bacteria (*p* < 0.01).

## Discussion

The cecum is thought to be the main site of fermentation. Cecal microbes play a crucial role in decomposing complex polysaccharides, such as uric acid, starch, and cellulose (Stanley et al., 2014). As chickens age, their gut microbial populations became more complex (Wielen et al., 2002). The α-diversity results showed that SF had a higher microbial community richness and unclassified bacteria than did WL. The complex microbiome indicated that SF had better adaptability and immunity to the environment. We found that the dominant phyla in WL and SF cecum were Bacteroidetes and Firmicutes. Consistent with our research, Pandit et al. (2018) found the same cecal microbiota in chickens, which typically accounted for more than 80% of the total number of microorganisms detected. Both Firmicutes and Bacteroidetes participate in fermentation, provide nutrition for the host (Gillilland et al., 2012), and are related to the metabolism of short-chain fatty acids (SCFAS), which not only inhibit the growth of pathogenic bacteria but also provide energy for the host (Józefiak et al., 2004). Studies have reported that a higher Firmicutes/Bacteroides ratio is associated with human obesity (Mariat et al., 2009), while the reverse is related to weight loss (Ley et al., 2005, 2006). Firmicutes promoted the synthesis of butyric as well as propionic acid and energy harvesting by improving lipid metabolism (Turnbaugh et al., 2008), while Bacteroidetes is usually associated with the degradation of polysaccharides (Degnan et al., 1997) and propionic acid synthesis (Polansky et al., 2016; Saxena et al., 2016) and promote energy metabolism by increasing carbohydrate metabolism (Kim J. K. et al., 2015). This was consistent with our study showing that the cecum of WL contained more Bacteroides and produced more propionic acid, which affected gluconeogenesis in the liver and glucose supply to the body. In chickens, Spirochetes can be potentially pathogenic, resulting in delayed and reduced egg production, slower growth, or diarrhea (Dwars et al., 2007). The disease can also be passed to offspring, making chicks weak, slow-growing, and having impaired gastrointestinal function (Smit et al., 2007). This was consistent with our study in that the content of Spirochetes in the cecum of SF was higher, which might be one of the reasons for their decline in egg production.

*Porphyromonadaceae*, *Veillonellaceae*, and *[Paraprevotellaceae]* were the dominant bacteria in SF. The *Veillonellaceae* family can utilize lactic acid and/or succinic acid to produce propionic acid, butyric acid, and/or valeric acid. *Veillonellaceae* was reported to produce high levels of SCFAS (acetate and propionate) (Lecomte et al., 2015). Cheng et al. (2018) found that the increased abundance of *Prevotellaceae* and *Veillonellaceae* caused glucose intolerance and insulin resistance in mice and that *Veillonellaceae* was related to serum insulin concentration. *Paraprevotellaceae* is associated with fatty acid synthesis and has positive health effects (Round and Mazmanian, 2009). Moreover, the abundance of *Paraprevotellaceae* and *Veillonellaceae* is involved in several functions and different pathways, including metabolic, protective, structural, and histological functions (Gallè et al., 2020). *Bacteroides* can produce carbohydrate metabolism-related enzymes, vitamins, glycans, and cofactor enzymes to promote food digestion (Karlsson et al., 2011). *Bacteroides* more prominently colonized chicken cecum’s with better growth performance, which indicated a closer relationship with glycan metabolism, while in chickens with worse growth performance, it was closely related to lipid metabolism (Cui et al., 2021). *Faecalibacterium* plays an important role in host physiology and health. Some studies have reported that *F. prausnitzii* has an anti-inflammatory activity and can secrete active anti-inflammatory substances, regulate its host immune response, and alleviate intestinal inflammation (Lopez-Siles et al., 2011; Heinken et al., 2014). The main metabolic end products of *Parabacteroides* are acetic acid and succinic acid, which are beneficial to the body. Wu et al. (2018) showed that the rate of increase of the body weight of high-fat diet mice was slower after oral administration of living *P. glosteinii* than that of the control group, and the amount of visceral fat, insulin resistance index, pro-inflammatory cytokines, serum endotoxin level, and intestinal permeability decreased. Therefore, *Parabacteroides* can enhance intestinal integrity, reduce inflammation, and potentially treat obesity (Wang et al., 2019). Based on the MetaCyc, KEGG, and COG databases, we found that metabolic pathways, such as glycan biosynthesis and metabolism, carbohydrate decomposition and metabolism, and glycan degradation, were highly enriched in WL, indicating that the bacterial community in WL was highly efficient in degrading carbohydrates and might produce more hydrolysates and energy. Compared to WL, SF were highly enriched in metabolic pathways, such as the TCA cycle, fatty acid and lipid metabolism, lipid transport and metabolism, and xenobiotics biodegradation, indicating that microorganisms in SF have an impact on lipid metabolism and immune metabolism.

The intestinal flora is an important metabolic “organ” in animals and can affect the overall metabolism of the host. There is a process of “co-metabolism” between the host and the flora. If the structure of intestinal flora changes, the physiological metabolism of the host would change correspondingly (Lee and Hase, 2014). The acetic acid and propionic acid contents were high in WL. The microorganisms in WL can fully digest the active ingredients in feed and degrade them to pyruvate, ultimately producing more volatile fatty acid. Acetic acid is a SCFA with the highest concentration in the body and is the center of the carbohydrate pathway. Acetic acid is an important source of host energy, providing approximately 10% of the total daily energy of the body (Cronin et al., 2021). Propionic acid is the main precursor of glucose synthesis in animals, which is conducive to the supply and transformation of energy and provides energy to the body. After being absorbed into the blood, propionic acid is catabolized and metabolized in the liver and participates in the process of reversing pyruvate into glucose, while possibly inhibiting the synthesis of fat (den Besten et al., 2013). Pyruvate enrichment increased the mutual conversion of fat, carbohydrate, and protein, thereby providing the body with more energy, which provided sufficient energy for egg laying. Acetic acid and propionic acid can also play antibacterial roles by promoting the release of host antimicrobial peptides. In addition, the content of deoxyuridine, L-proline, and spermidine in WL was increased, and the ABC transporter pathway enriched in these metabolites played an important role as a multifunctional transmembrane protein in cell osmotic pressure regulation (Jaskulak et al., 2020). SF contained high levels of 6-hydroxypseudooxynicotine, which is involved in the metabolism of nicotinic acid, a B vitamin derived from the synthesis of gut microbes and directly supplied to feed, and was essential for animal growth and development. Nicotinate and nicotinamide participated in lipid metabolism (Kamanna and Kashyap, 2008) and reduced abdominal fat (Jiang et al., 2014), which may increase lipid degradation and metabolism in chickens.

Melanin biosynthesis and metabolism in SF are important pathways related to immunity. Melanin had antioxidant (Liu et al., 2011), anti-virus (Manning et al., 2003), and gastrointestinal health- modulation effects (El-Obeid et al., 2016). In addition, melanin can also interact with the immune system in a variety of ways, such as by improving the efficacy of antibiotics (Fukuda and Sasaki, 1990), inhibiting inflammation (Nosanchuk and Casadevall, 2003), and enhancing various immune parameters (Pugh et al., 2005). *CRP* was highly expressed in SF, which could play an important role in the natural immunity of the body by enhancing phagocytosis and eliminating necrotic tissue cells (Sproston and Ashworth, 2018). The PPAR metabolism pathway is involved in lipogenesis (Cristancho and Lazar, 2011), fatty acid metabolism (Grygiel-Gorniak, 2014), and immune response (Christofides et al., 2021), and maintains metabolic homeostasis. *ACSBG1* was highly expressed in SF and participates in the PPAR metabolic pathway (Gharib-Naseri et al., 2021), which affects fatty acid metabolism and degradation in animals. *AKR1D1* was highly expressed in WL, promoting bile acid metabolism (Chaudhry et al., 2013) and follicular growth (Zhang et al., 2016), and *EDA2R*, a significantly upregulated gene in WL, was confirmed to be related to egg production (Chen et al., 2021), which may promote egg production in WL. The expression level of *PFKFB4* increased in WL, participating in glucose metabolism and providing energy to the body (Shen et al., 2021), which might be conducive to egg production. *MGAT5B* participates in the metabolic pathway of N-Glycan biosynthesis, and N-glycation modification plays an important role in protein folding, transportation, and other processes (Liu et al., 2014).

The cecal microbes in chicken are closely related to the feeding, health status, and metabolism of their host. However, few studies have explored the relationship among microbial taxa, metabolites, and gene transcription levels. Therefore, how cecal microorganisms interact with their metabolites and gene transcription levels at a deeper taxonomic level remains unclear. In this study, the relative abundances of microorganisms and host cecum genes were analyzed for their correlations with cecal metabolites. We found that the metabolites acetate, propanoate, L-proline, and spermidine were positively correlated with *Bacteroides* in WL. *Bacteroides* played a major role in glycosaminoglycan biosynthesis, propanoate metabolism, and the ABC transporter pathway. Adamberg et al. (2014) showed that acetic acid and propionic acid are fermentation products of *Bacteroides*. Acetate and propionate, the metabolites of *Bacteroides*, had positive effects on glycolysis and propionate metabolism. Genes related to acetic acid and propionic acid were significantly upregulated in the cecum transcriptome. The increase in acetic acid and propionic acid contents enhanced carbohydrate metabolism and provided more energy for the body, which may support an increase in egg production. SF and WL had different dominant bacteria, and the unique bacteria in SF impacted the metabolite acetic acid, inhibiting the production of volatile fatty acids, and had no positive effect on egg production. The metabolites volicitin, 6-hydroxypseudooxynicotine, and D-cathinone were positively correlated with *Parabacteroides* and *Veillonellaceae* in SF. There was a significant positive correlation between *Parabacteroides* and volicitin, which is a metabolite of the α-linolenic acid metabolism pathway, increasing lipid deposition, which occurred in the submucosa of SF cecum. RNA-seq results also showed that melanin production, lipid metabolism, and immune-related genes were significantly upregulated, regulating lipid metabolism, participating in the immune response, and maintaining homeostasis.

## Conclusion

In this study, multi-omics was used to comprehensively analyze differences in cecal metabolism between SF and WL under the same environmental conditions. The high enrichment of *Bacteroidetes* in WL supplied more amino acids and energy to the body and provided the basis for the sustained high level of egg production. *Veillonellaceae* and *Parabacteroides* were enriched in SF, which regulated lipid metabolism and participated in the immune response. This study provides insights into strategies for altering the cecal microbiota to achieve higher egg production and better immunity through feeding management or genetic selection.

## Data availability statement

The datasets presented in this study can be found in online repositories. The names of the repository/repositories and accession number(s) can be found in the article/Supplementary material.

## Ethics statement

This animal study was reviewed and approved by the Laboratory Animal Welfare Experiment License from China Agricultural University was obtained (permit number: SKLAB-2012-04-07). All experiments were approved by the Committee on the Animal Experimental Ethical Inspection of China Agricultural University (issue number: AW32802202-1-1).

## Author contributions

XD and XY conceived the project and designed the experiments. XY, YT, YM, and JL collected the samples for 16S rDNA gene amplicon sequencing, untargeted metabolomics and RNA-seq, and determined phenotypic index. XY and DH planned the data analyses and oil red O staining. XY wrote the manuscript. DH and XD revised the manuscript. XD supervised the work and edited manuscripts. All authors contributed to the article and approved the submitted version.

## Footnotes

1
https://docs.qiime2.org/2019.4/tutorials/

2
http://huttenhower.sph.harvard.edu/lefse/

3
https://metacyc.org/

4
https://www.kegg.jp/

5
https://www.ncbi.nlm.nih.gov/COG/

6
https://www.genescloud.cn/login

7
http://metorigin.met-bioinformatics.cn/

## Supplementary material

Supplementary Figure 1 Response sequence test analysis and volcano plot of differential metabolite in positive ion and negative ion mode.

Supplementary Figure 2 Bar plot and Venn diagram of the number of metabolites in WL and SF separately in different categories.

Supplementary Figure 3 The metabolic pathways of bacterial metabolism and cometabolism pathways with the host.

Supplementary Figure 4 The Spearman correlation analysis between bacteria at genus level and metabolites.

Supplementary Figure 5 The BIO-Sankey network for several metabolic reactions in significantly different metabolism pathways.
