# Supplementary material for: Cecum microbiome and metabolism characteristics of Silky Fowl and White Leghorn chicken in late laying stages
Source: Front Microbiol. 2022 Oct 20;13:984654. doi: 10.3389/fmicb.2022.984654 (PMC9633115; doi:10.3389/fmicb.2022.984654)
Supplement: Supplementary Figure 1 — Response sequence test analysis and volcano plot of differential metabolite in positive ion and negative ion mode. [file Data_Sheet_1.PDF]

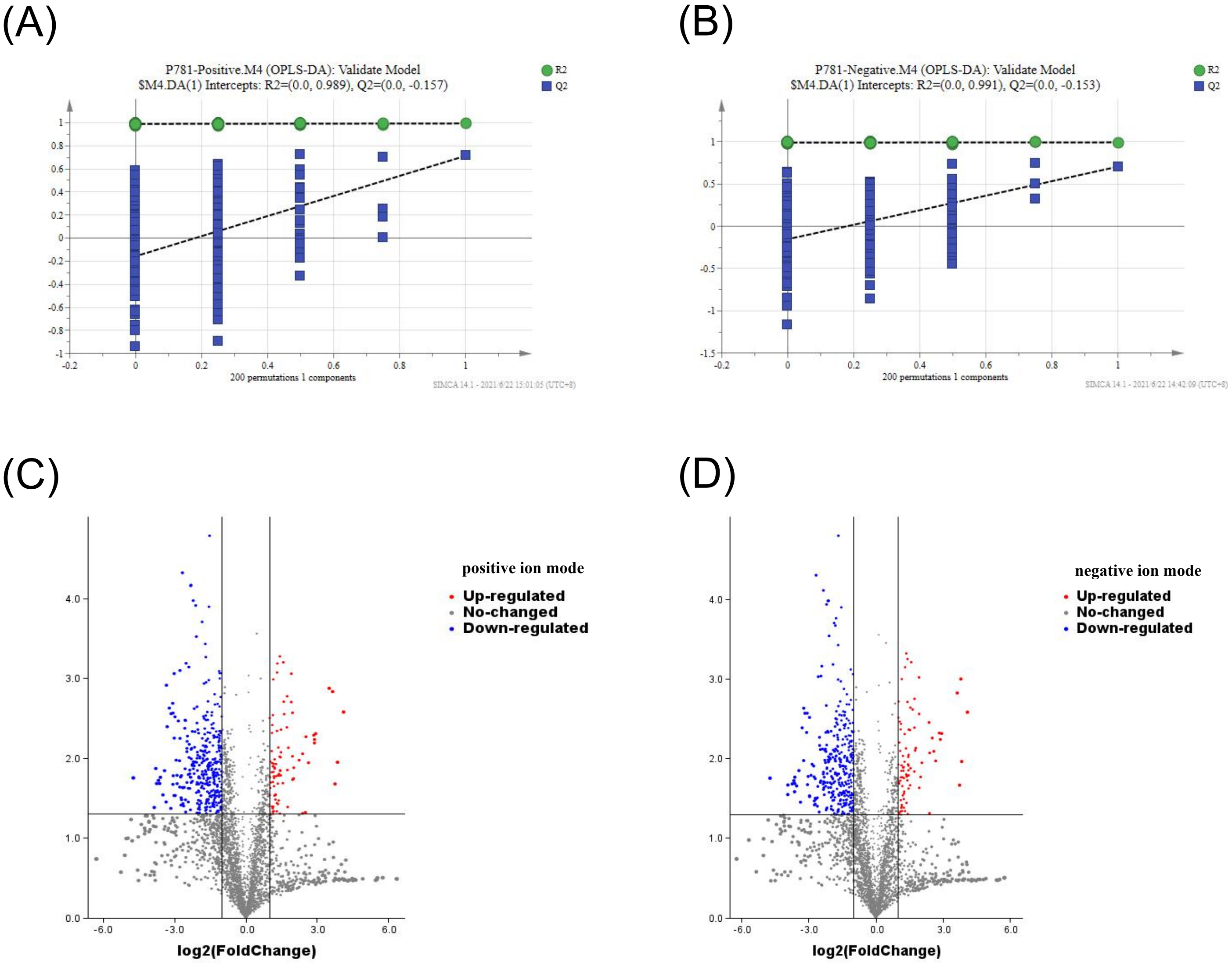

Supplementary Figure S1 Response sequence test analysis in (A) positive ion (ESI+) and (B) negative ion (ESI-) mode. The volcano plot of differential metabolite screening in positive ion (C) and negative ion mode (D).

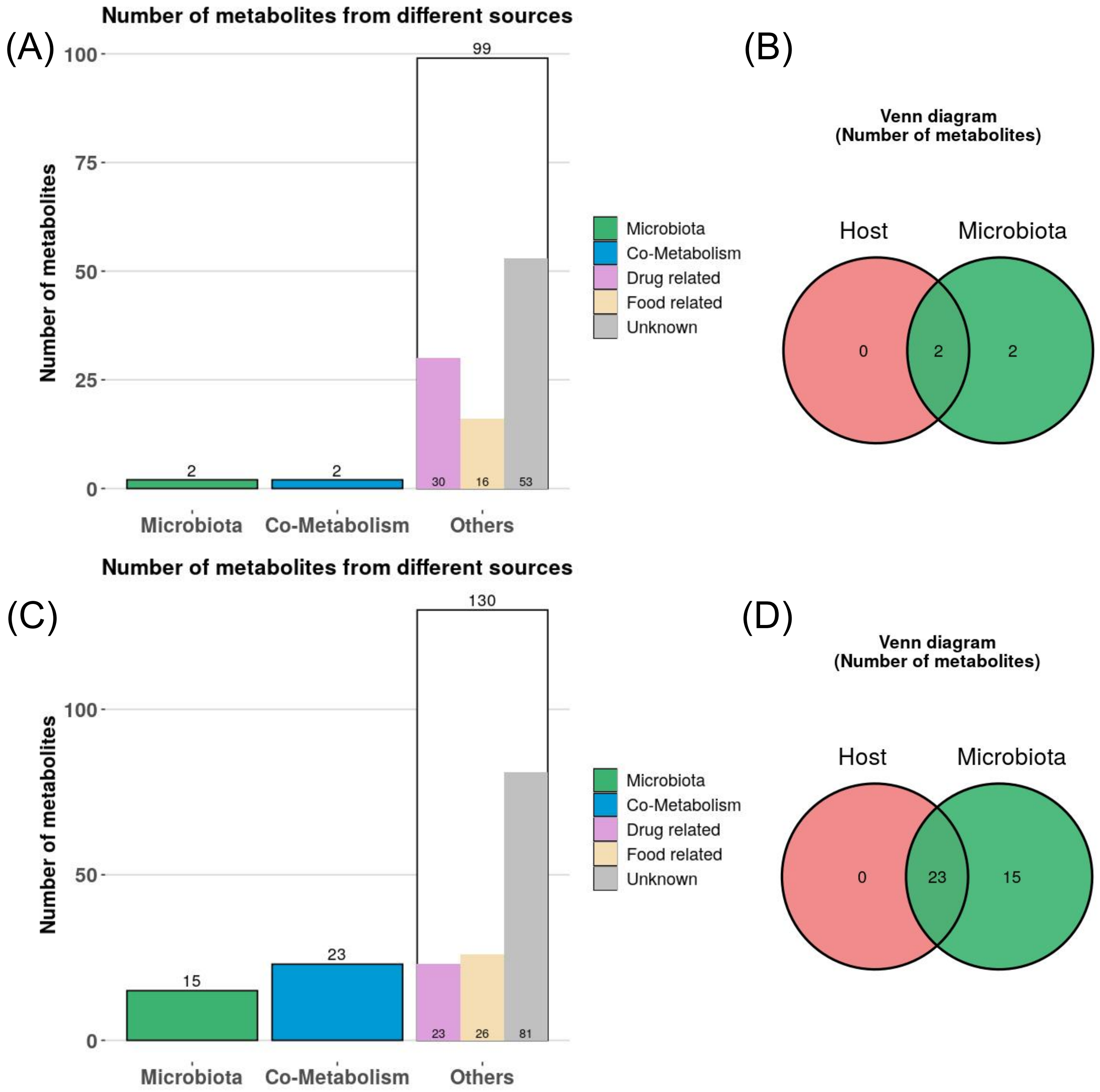

Supplementary Figure S2 Bar plot and Venn diagram of the number of metabolites in WL and SF separately in different categories. (A) Venn diagram of the number of metabolic pathways in microbial community of SF. (B) Venn diagram of the number of metabolites in bacterial communities of SF. (C) Venn diagram of the number of metabolic pathways in microbial community of WL. (D) Venn diagram of the number of metabolites in bacterial communities of WL.

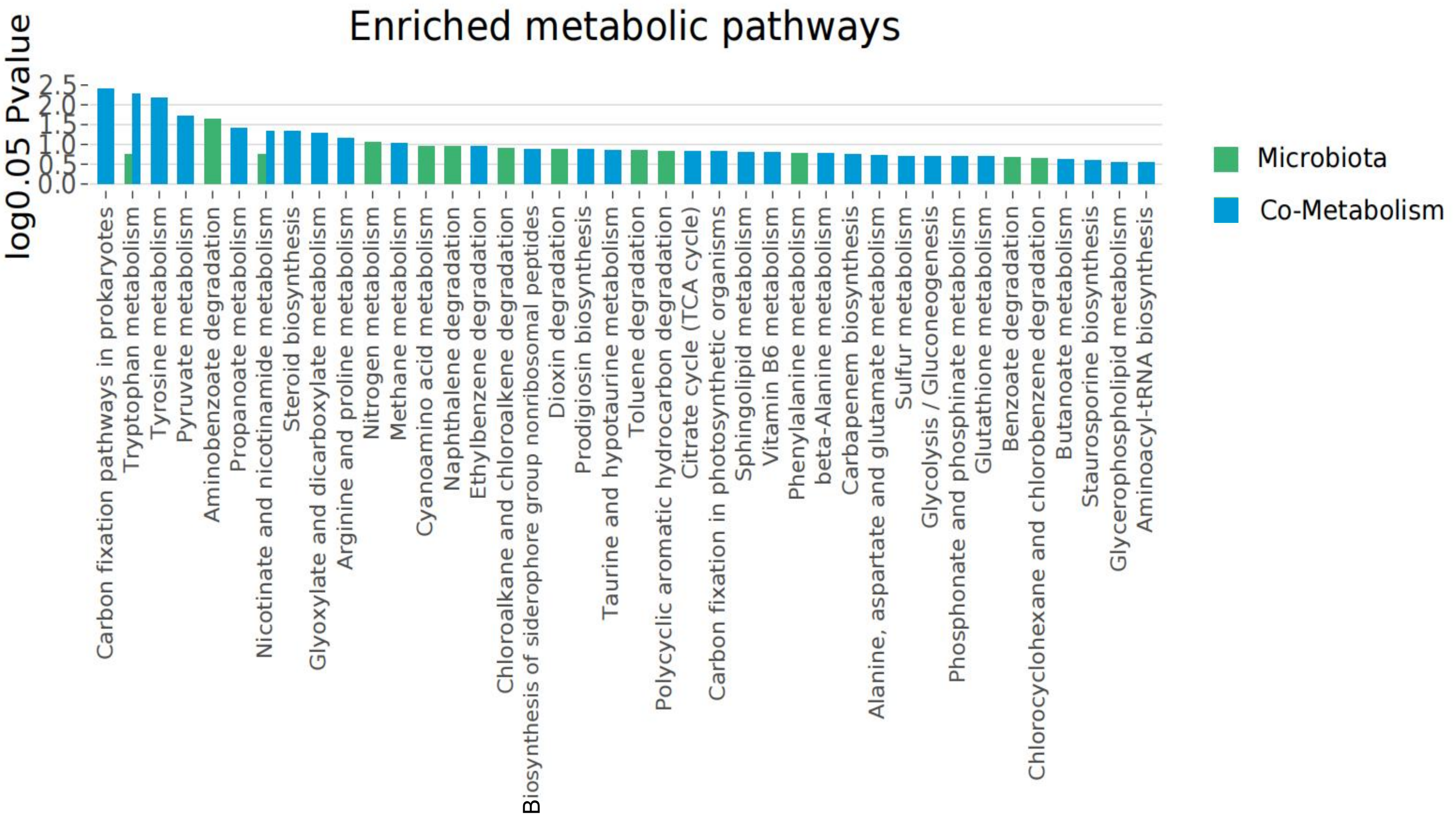

Supplementary Figure S3 The metabolic pathways of bacterial metabolism and co-metabolism pathways with the host.

The metabolic pathway enrichment analysis performed according to different categories of metabolites: metabolites belonging to bacteria, or the co-metabolism between host and thebacteria.

Note: P values are calculated using the hypergeometric test. The Y-axis indicates the log 0.05 transformation of P values. Metabolic pathways with log 0.05 P values greater than 1 are considered statistically significant.

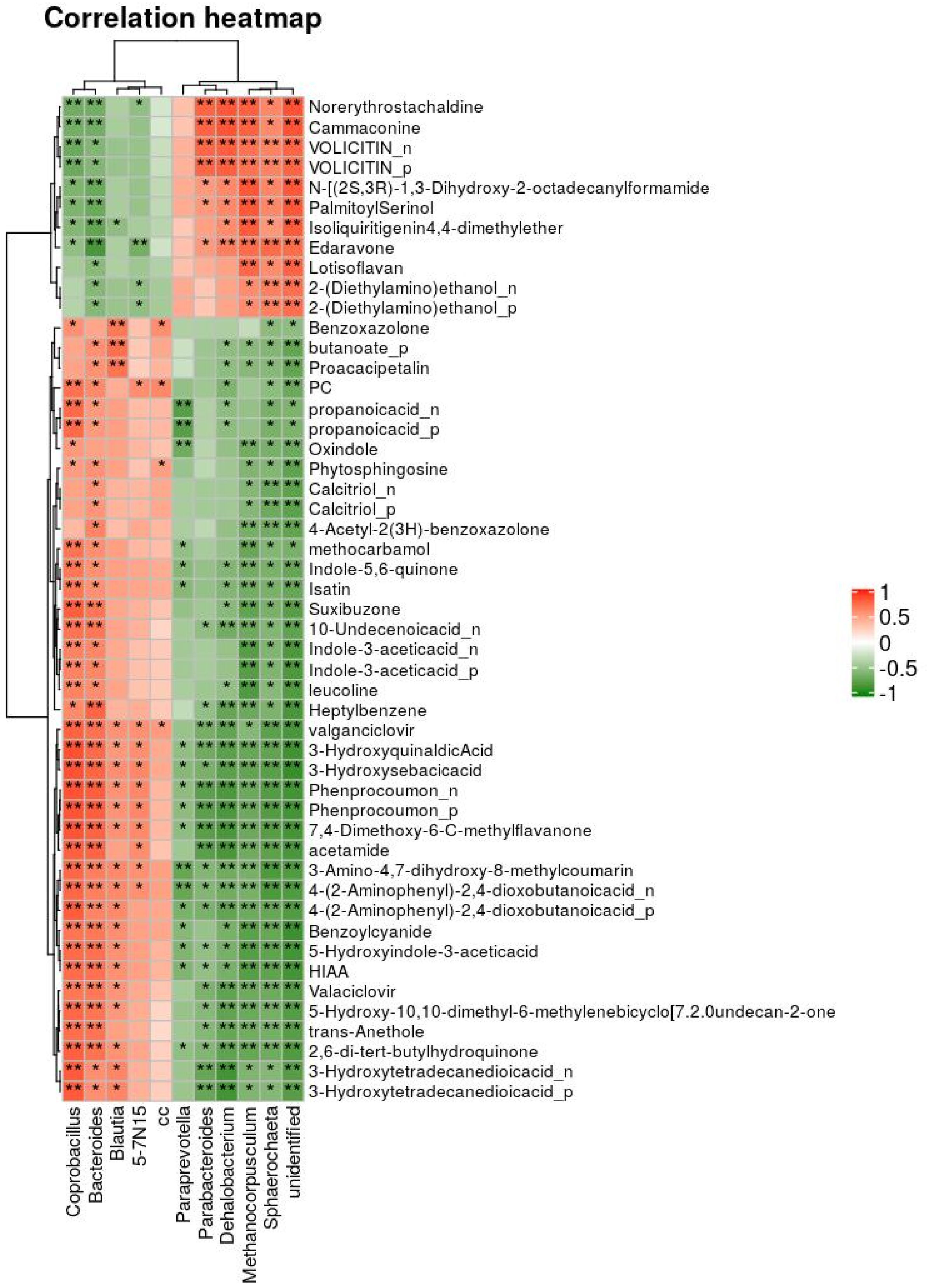

Supplementary Figure S4 The Spearman correlation analysis between bacteria at genus level and metabolites.
Asterisk coding is indicated in the Figure Legends as *P < 0.05; **P<0.01; ***P < 0.001.

(A)

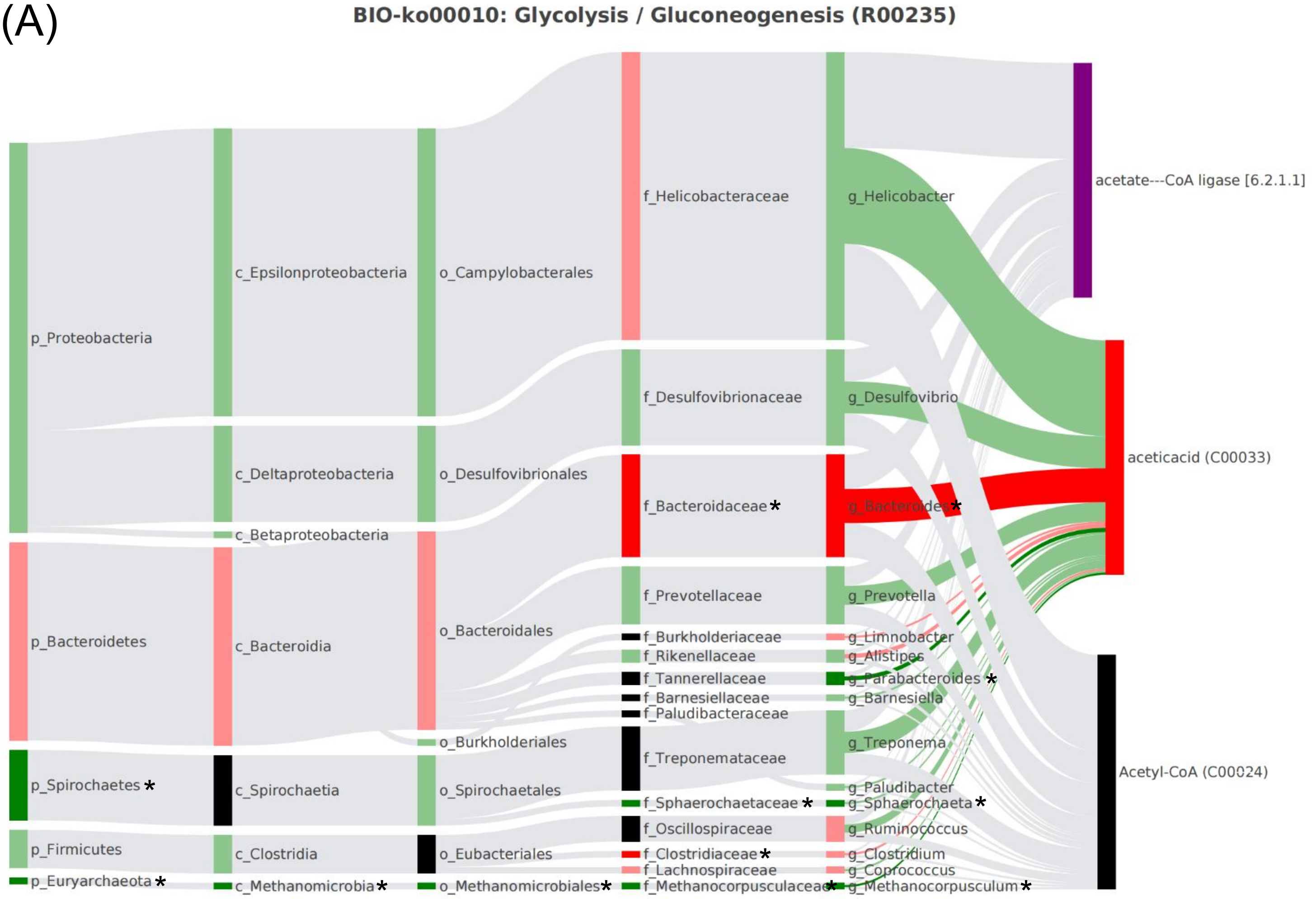
BIO-ko00010: Glycolysis / Gluconeogenesis (R00235)
p_Proteobacteria
p_Bacteroidetes
p_Spirochaetes *
p_Firmicutes
p_Euryarchaeota *
c_Epsilonproteobacteria
c_Deltaproteobacteria
c_Betaproteobacteria
c_Bacteroidia
c_Spirochaetia
c_Clostridia
c_Methanomicrobia *
o_Campylobacterales
o_Desulfovibrionales
o_Bacteroidales
o_Burkholderiales
o_Spirochaetales
o_Eubacteriales
o_Methanomicrobiales *
f_Helicobacteraceae
f_Desulfovibrionaceae
f_Bacteroidaceae *
f_Prevotellaceae
f_Burkholderiaceae
f_Rikenellaceae
f_Tannerellaceae
f_Barnesiellaceae
f_Paludibacteraceae
f_Treponemataceae
f_Sphaerochaetaceae *
f_Oscillospiraceae
f_Clostridiaceae *
f_Lachnospiraceae
f_Methanocorpusculaceae *
g_Helicobacter
g_Desulfovibrio
g_Bacteroides *
g_Prevotella
g_Limnobacter
g_Alistipes
g_Parabacteroides *
g_Barnesiella
g_Treponema
g_Paludibacter
g_Sphaerochaeta *
g_Ruminococcus
g_Clostridium
g_Coprococcus
g_Methanocorpusculum *
acetate---CoA ligase [6.2.1.1]
aceticacid (C00033)
Acetyl-CoA (C00024)

(B)

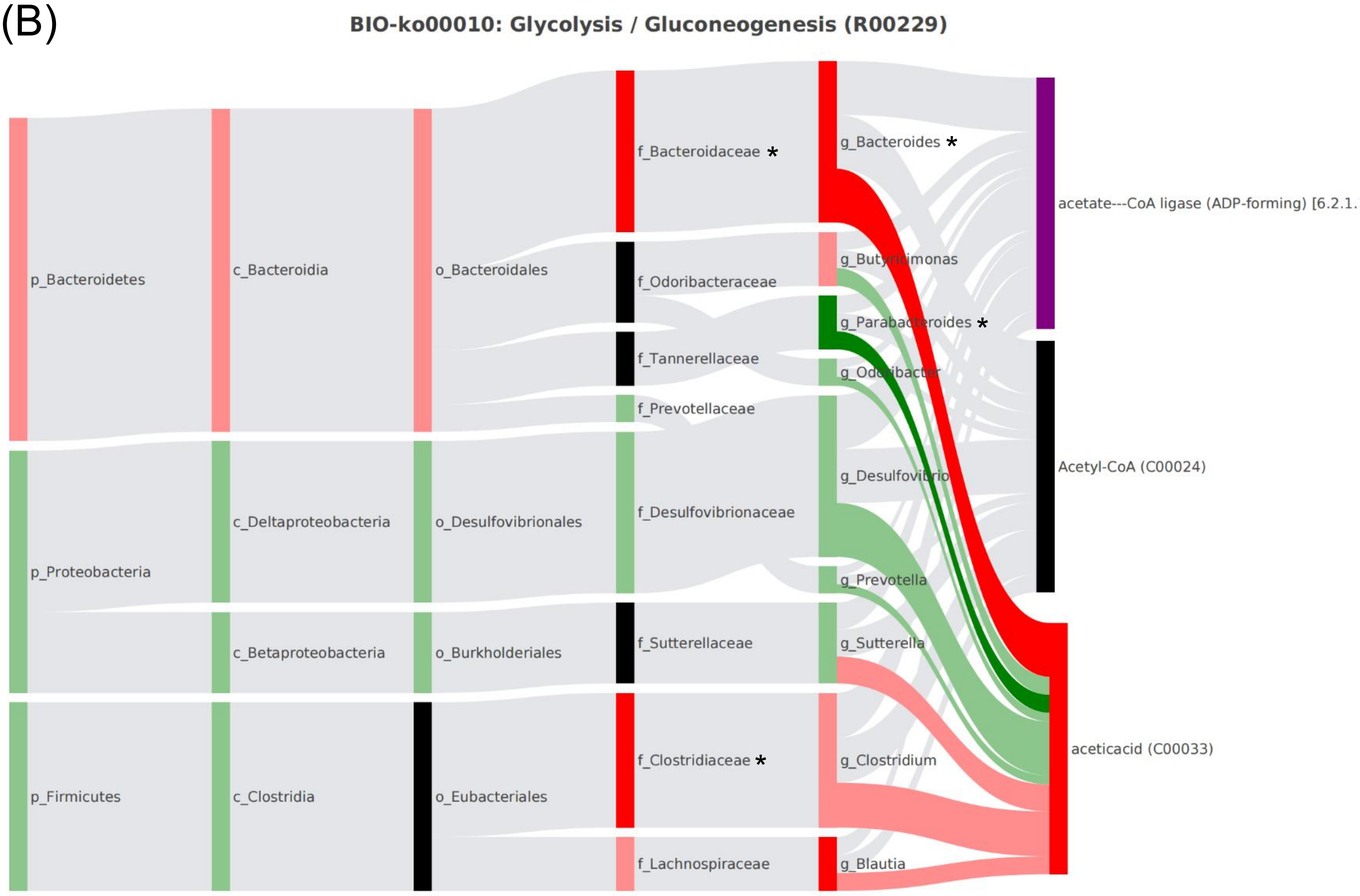
BIO-ko00010: Glycolysis / Gluconeogenesis (R00229)
p_Bacteroidetes
p_Proteobacteria
p_Firmicutes
c_Bacteroidia
c_Deltaproteobacteria
c_Betaproteobacteria
c_Clostridia
o_Bacteroidales
o_Desulfovibrionales
o_Burkholderiales
o_Eubacteriales
f_Bacteroidaceae *
f_Odoribacteraceae
f_Tannerellaceae
f_Prevotellaceae
f_Desulfovibrionaceae
f_Sutterellaceae
f_Clostridiaceae *
f_Lachnospiraceae
g_Bacteroides *
g_Butyricimonas
g_Parabacteroides *
g_Odoribacter
g_Desulfovibrio
g_Prevotella
g_Sutterella
g_Clostridium
g_Blautia
acetate---CoA ligase (ADP-forming) [6.2.1.
Acetyl-CoA (C00024)
aceticacid (C00033)

(C)

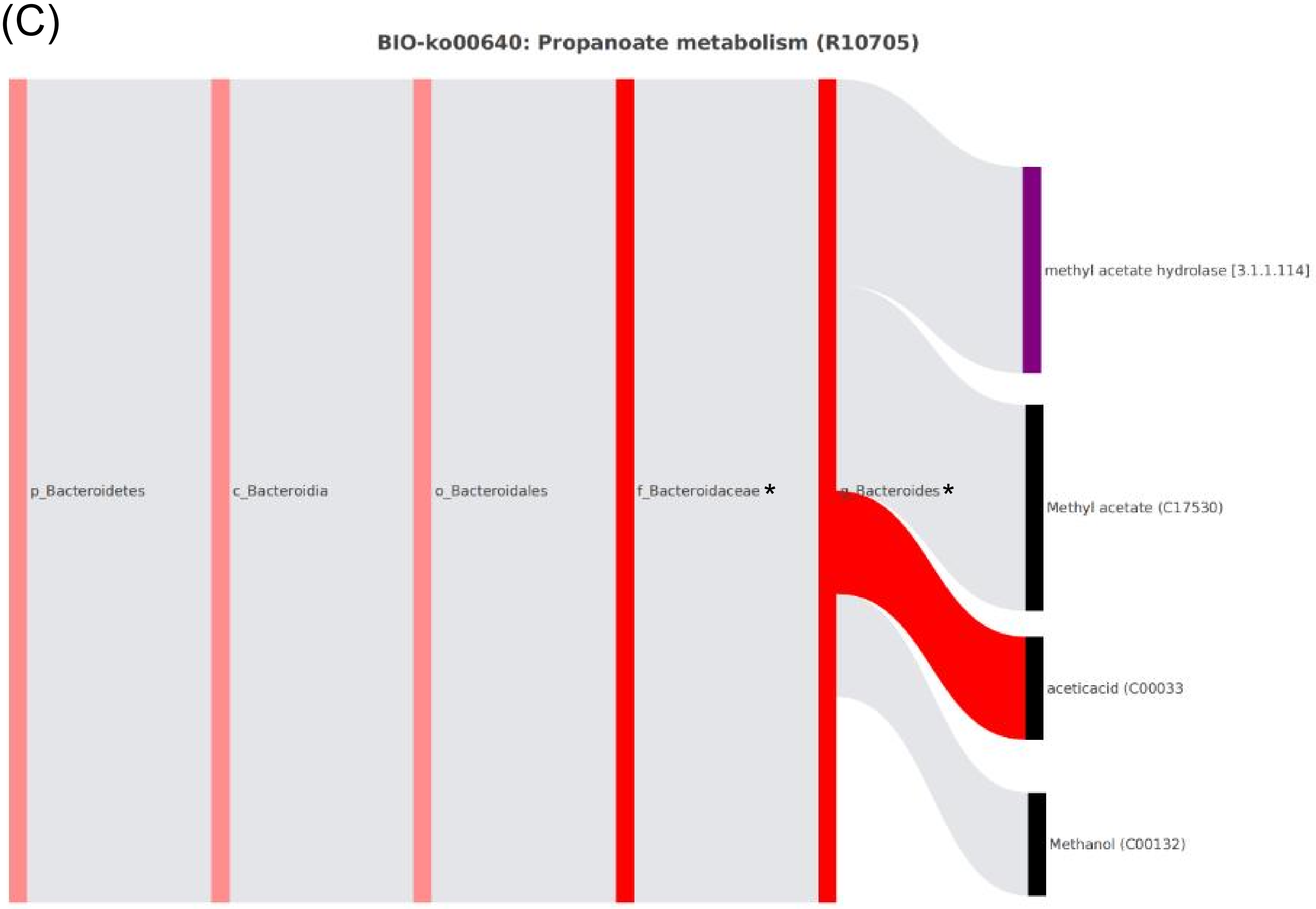
BIO-ko00640: Propanoate metabolism (R10705)
p_Bacteroidetes
c_Bacteroidia
o_Bacteroidales
f_Bacteroidaceae *
g_Bacteroides *
methyl acetate hydrolase [3.1.1.114]
Methyl acetate (C17530)
aceticacid (C00033
Methanol (C00132)

(D)

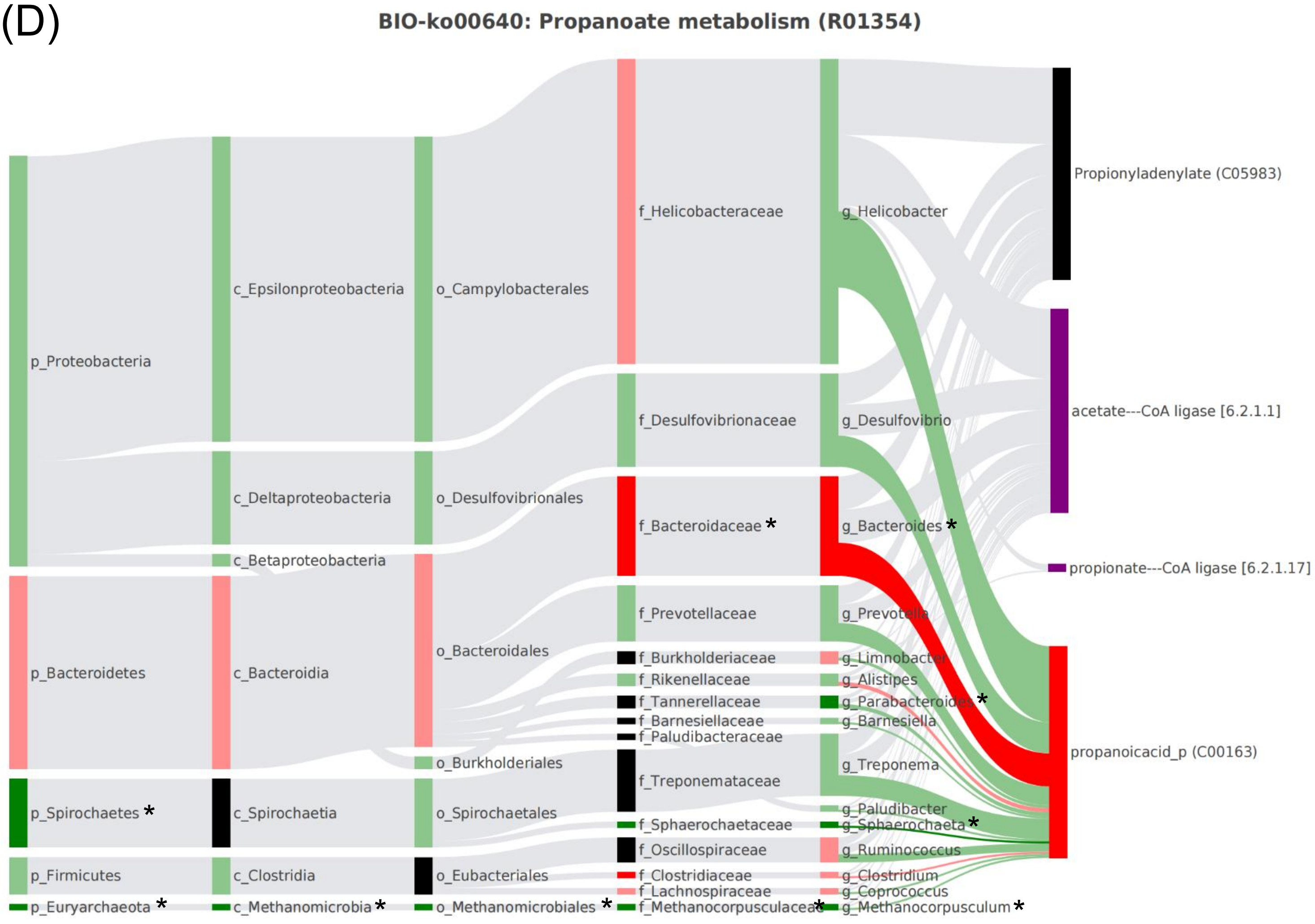
BIO-ko00640: Propanoate metabolism (R01354)
p_Proteobacteria
p_Bacteroidetes
p_Spirochaetes *
p_Firmicutes
p_Euryarchaeota *
c_Epsilonproteobacteria
c_Deltaproteobacteria
c_Betaproteobacteria
c_Bacteroidia
c_Spirochaetia
c_Clostridia
c_Methanomicrobia *
o_Campylobacterales
o_Desulfovibrionales
o_Bacteroidales
o_Burkholderiales
o_Spirochaetales
o_Eubacteriales
o_Methanomicrobiales *
f_Helicobacteraceae
f_Desulfovibrionaceae
f_Bacteroidaceae *
f_Prevotellaceae
f_Burkholderiaceae
f_Rikenellaceae
f_Tannerellaceae
f_Barnesiellaceae
f_Paludibacteraceae
f_Treponemataceae
f_Sphaerochaetaceae
f_Oscillospiraceae
f_Clostridiaceae
f_Lachnospiraceae
f_Methanocorpusculaceae *
g_Helicobacter
g_Desulfovibrio
g_Bacteroides *
g_Prevotella
g_Limnobacter
g_Alistipes
g_Parabacteroides *
g_Barnesiella
g_Treponema
g_Paludibacter
g_Sphaerochaeta *
g_Ruminococcus
g_Clostridium
g_Coprococcus
g_Methanocorpusculum *
Propionyladenylate (C05983)
acetate---CoA ligase [6.2.1.1]
propionate---CoA ligase [6.2.1.17]
propanoicacid_p (C00163)

(E)

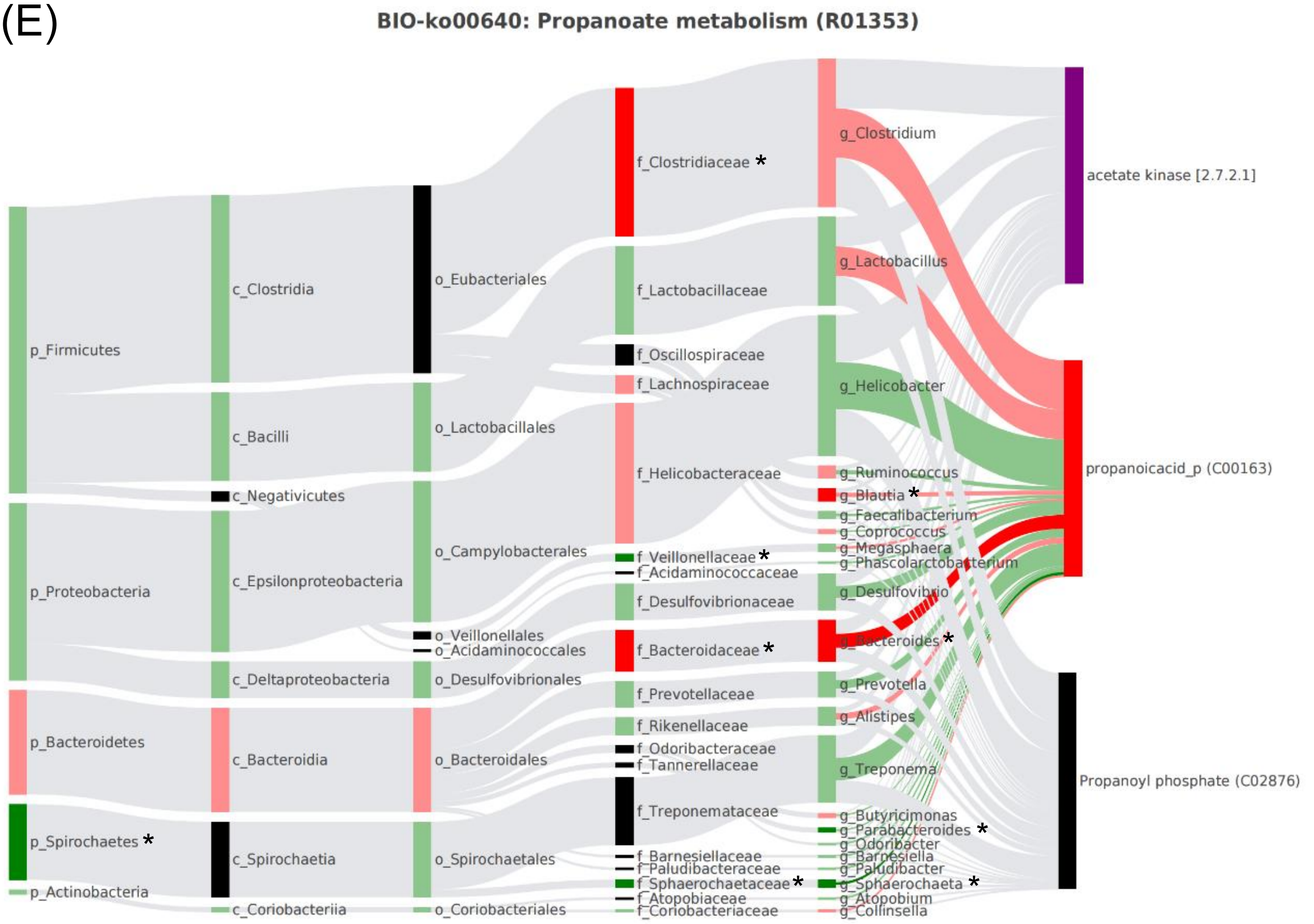
BIO-ko00640: Propanoate metabolism (R01353)
p_Firmicutes
p_Proteobacteria
p_Bacteroidetes
p_Spirochaetes *
p_Actinobacteria
c_Clostridia
c_Bacilli
c_Negativicutes
c_Epsilonproteobacteria
c_Deltaproteobacteria
c_Bacteroidia
c_Spirochaetia
c_Coriobacteriia
o_Eubacteriales
o_Lactobacillales
o_Campylobacterales
o_Veillonellales
o_Acidaminococcales
o_Desulfovibrionales
o_Bacteroidales
o_Spirochaetales
o_Coriobacteriales
f_Clostridiaceae *
f_Lactobacillaceae
f_Oscillospiraceae
f_Lachnospiraceae
f_Helicobacteraceae
f_Veillonellaceae *
f_Acidaminococcaceae
f_Desulfovibrionaceae
f_Bacteroidaceae *
f_Prevotellaceae
f_Rikenellaceae
f_Odoribacteraceae
f_Tannerellaceae
f_Treponemataceae
f_Barnesiellaceae
f_Paludibacteraceae
f_Sphaerochaetaceae *
f_Atopobiaceae
f_Coriobacteriaceae
g_Clostridium
g_Lactobacillus
g_Helicobacter
g_Ruminococcus
g_Blautia *
g_Faecalibacterium
g_Coprococcus
g_Megasphaera
g_Phascolarctobacterium
g_Desulfovibrio
g_Bacteroides *
g_Prevotella
g_Alistipes
g_Treponema
g_Butyricimonas
g_Parabacteroides *
g_Odoribacter
g_Barnesiella
g_Paludibacter
g_Sphaerochaeta *
g_Atopobium
g_Collinsella
acetate kinase [2.7.2.1]
propanoicacid_p (C00163)
Propanoyl phosphate (C02876)

(F)

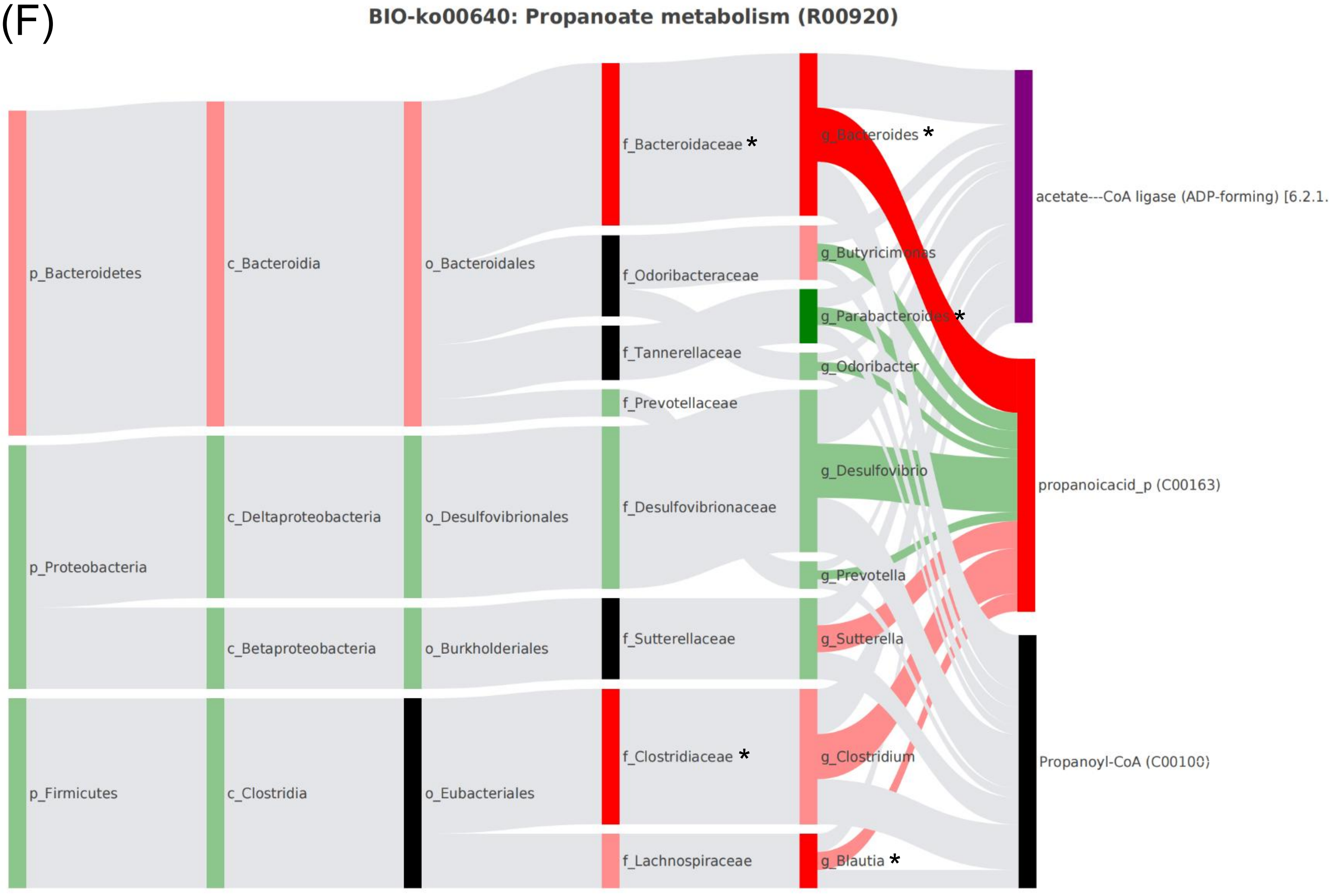
BIO-ko00640: Propanoate metabolism (R00920)
p_Bacteroidetes
p_Proteobacteria
p_Firmicutes
c_Bacteroidia
c_Deltaproteobacteria
c_Betaproteobacteria
c_Clostridia
o_Bacteroidales
o_Desulfovibrionales
o_Burkholderiales
o_Eubacteriales
f_Bacteroidaceae *
f_Odoribacteraceae
f_Tannerellaceae
f_Prevotellaceae
f_Desulfovibrionaceae
f_Sutterellaceae
f_Clostridiaceae *
f_Lachnospiraceae
g_Bacteroides *
g_Butyricimonas
g_Parabacteroides *
g_Odoribacter
g_Desulfovibrio
g_Prevotella
g_Sutterella
g_Clostridium
g_Blautia *
acetate---CoA ligase (ADP-forming) [6.2.1.
propanoicacid_p (C00163)
Propanoyl-CoA (C00100)

(G)

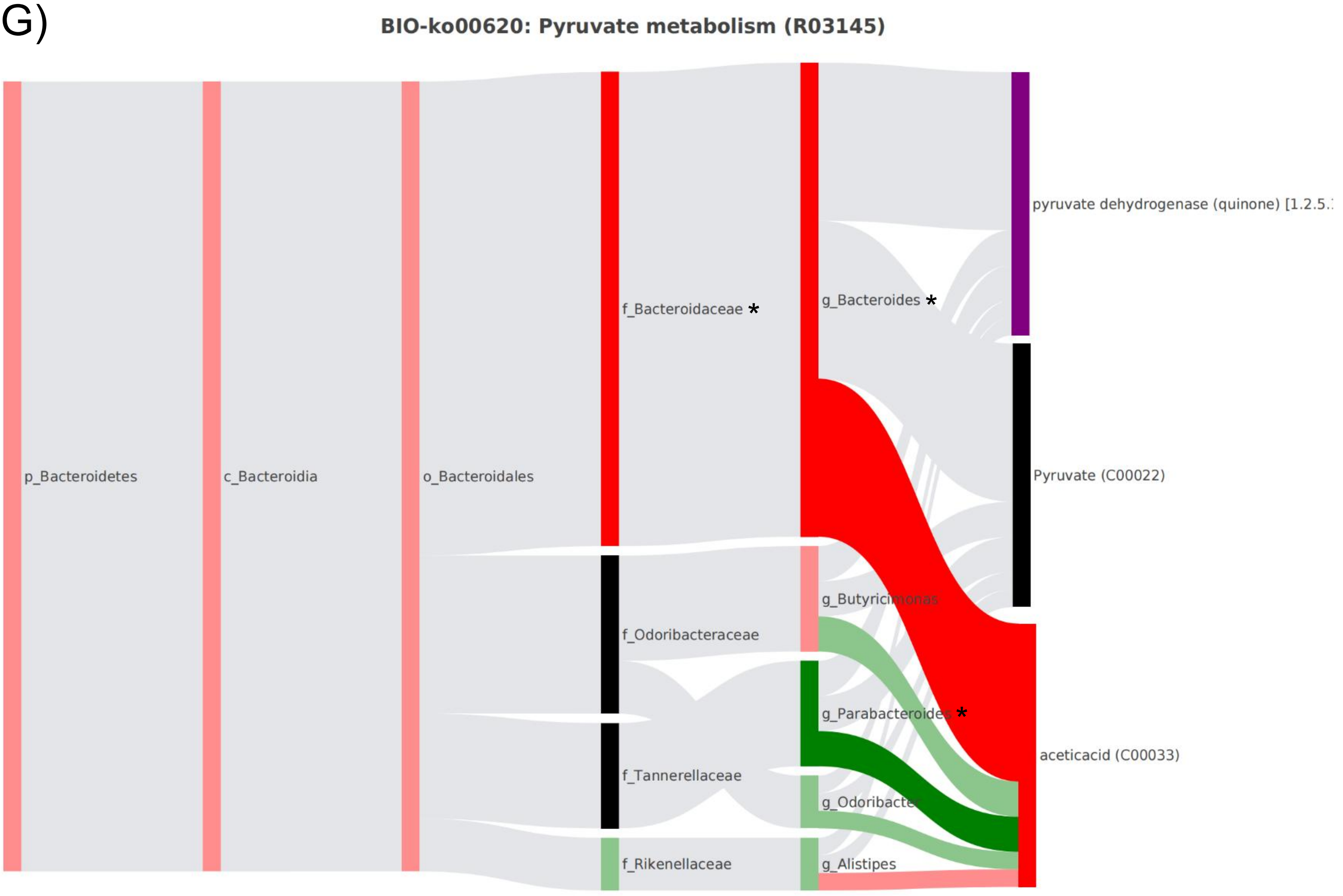
BIO-ko00620: Pyruvate metabolism (R03145)
p_Bacteroidetes
c_Bacteroidia
o_Bacteroidales
f_Bacteroidaceae *
f_Odoribacteraceae
f_Tannerellaceae
f_Rikenellaceae
g_Bacteroides *
g_Butyricimonas
g_Parabacteroides *
g_Odoribacter
g_Alistipes
pyruvate dehydrogenase (quinone) [1.2.5.1]
Pyruvate (C00022)
aceticacid (C00033)

(H)

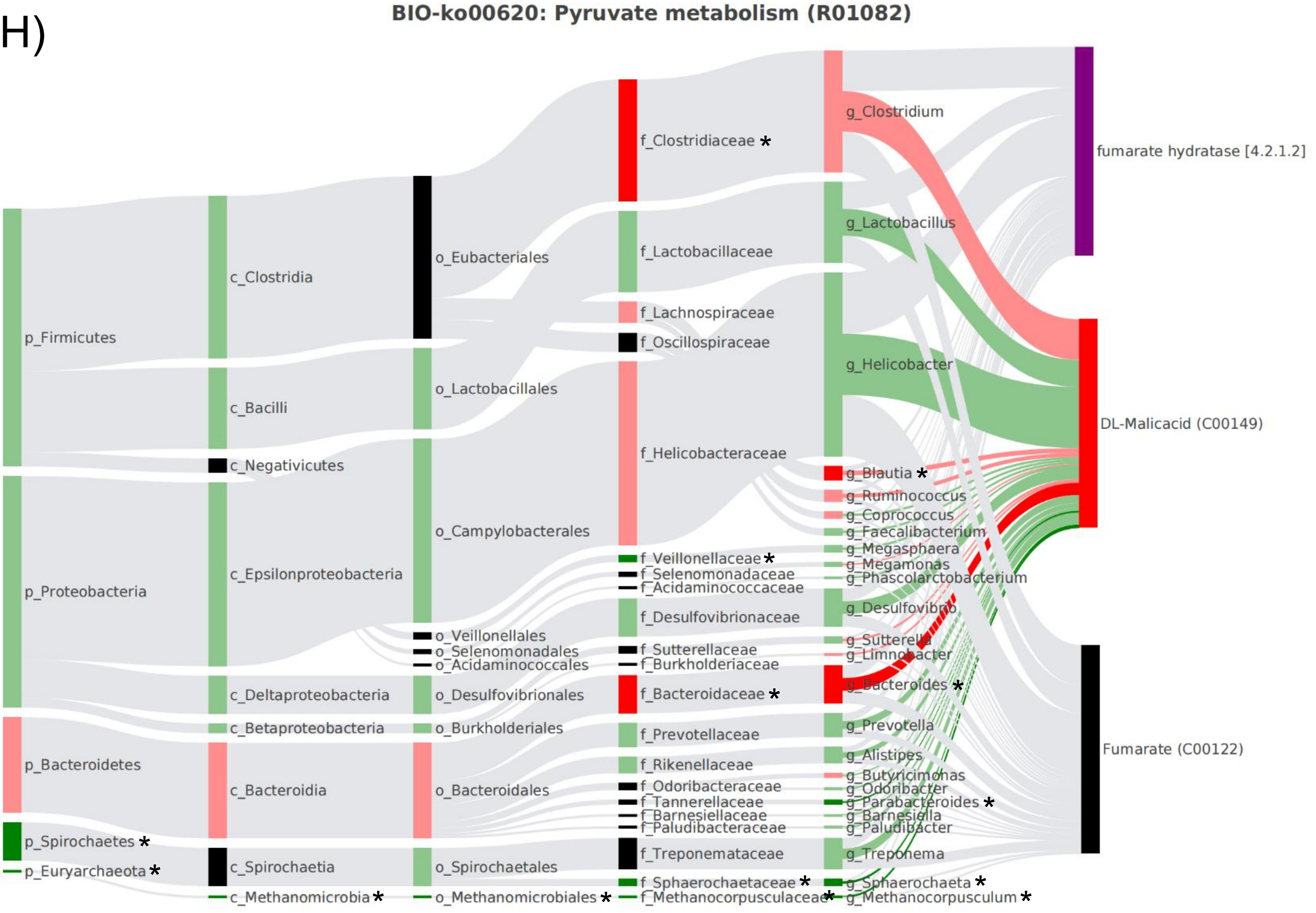
BIO-ko00620: Pyruvate metabolism (R01082)
p_Firmicutes
p_Proteobacteria
p_Bacteroidetes
p_Spirochaetes *
p_Euryarchaeota *
c_Clostridia
c_Bacilli
c_Negativicutes
c_Epsilonproteobacteria
c_Deltaproteobacteria
c_Betaproteobacteria
c_Bacteroidia
c_Spirochaetia
c_Methanomicrobia *
o_Eubacteriales
o_Lactobacillales
o_Campylobacterales
o_Veillonellales
o_Selenomonadales
o_Acidaminococcales
o_Desulfovibrionales
o_Burkholderiales
o_Bacteroidales
o_Spirochaetales
o_Methanomicrobiales *
f_Clostridiaceae *
f_Lactobacillaceae
f_Lachnospiraceae
f_Oscillospiraceae
f_Helicobacteraceae
f_Veillonellaceae *
f_Selenomonadaceae
f_Acidaminococcaceae
f_Desulfovibrionaceae
f_Sutterellaceae
f_Burkholderiaceae
f_Bacteroidaceae *
f_Prevotellaceae
f_Rikenellaceae
f_Odoribacteraceae
f_Tannerellaceae
f_Barnesiellaceae
f_Paludibacteraceae
f_Treponemataceae
f_Sphaerochaetaceae *
f_Methanocorpusculaceae *
g_Clostridium
g_Lactobacillus
g_Helicobacter
g_Blautia *
g_Ruminococcus
g_Coprococcus
g_Faecalibacterium
g_Megasphaera
g_Megamonas
g_Phascolarctobacterium
g_Desulfovibrio
g_Sutterella
g_Limnobacter
g_Bacteroides *
g_Prevotella
g_Alistipes
g_Butyricimonas
g_Odoribacter
g_Parabacteroides *
g_Barnesiella
g_Paludibacter
g_Treponema
g_Sphaerochaeta *
g_Methanocorpusculum *
fumarate hydratase [4.2.1.2]
DL-Malicacid (C00149)
Fumarate (C00122)

(I)

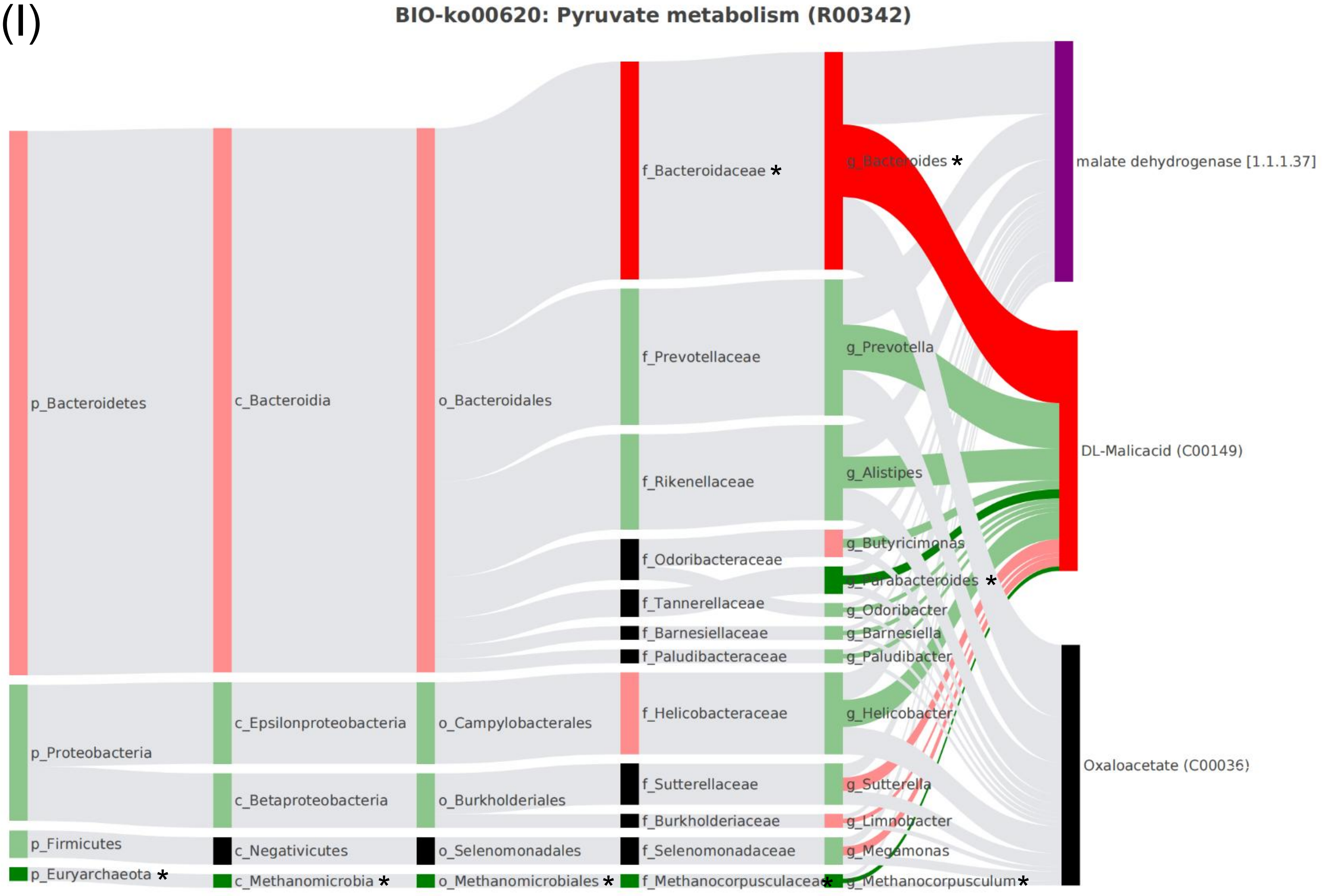
BIO-ko00620: Pyruvate metabolism (R00342)
p_Bacteroidetes
p_Proteobacteria
p_Firmicutes
p_Euryarchaeota *
c_Bacteroidia
c_Epsilonproteobacteria
c_Betaproteobacteria
c_Negativicutes
c_Methanomicrobia *
o_Bacteroidales
o_Campylobacterales
o_Burkholderiales
o_Selenomonadales
o_Methanomicrobiales *
f_Bacteroidaceae *
f_Prevotellaceae
f_Rikenellaceae
f_Odoribacteraceae
f_Tannerellaceae
f_Barnesiellaceae
f_Paludibacteraceae
f_Helicobacteraceae
f_Sutterellaceae
f_Burkholderiaceae
f_Selenomonadaceae
f_Methanocorpusculaceae *
g_Bacteroides *
g_Prevotella
g_Alistipes
g_Butyricimonas
g_Parabacteroides *
g_Odoribacter
g_Barnesiella
g_Paludibacter
g_Helicobacter
g_Sutterella
g_Limnobacter
g_Megamonas
g_Methanocorpusculum *
malate dehydrogenase [1.1.1.37]
DL-Malicacid (C00149)
Oxaloacetate (C00036)

(J)

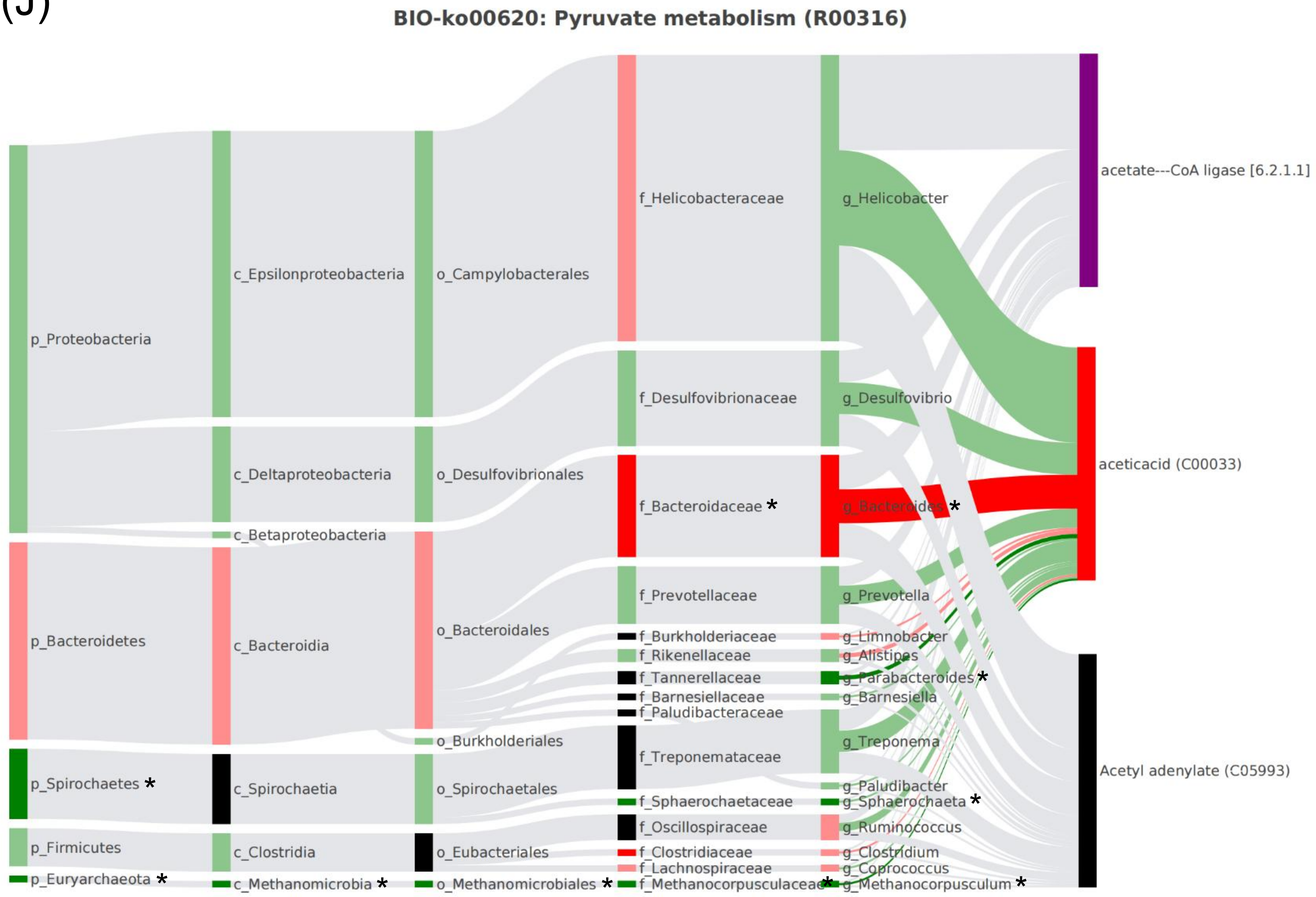
BIO-ko00620: Pyruvate metabolism (R00316)
p_Proteobacteria
p_Bacteroidetes
p_Spirochaetes *
p_Firmicutes
p_Euryarchaeota *
c_Epsilonproteobacteria
c_Deltaproteobacteria
c_Betaproteobacteria
c_Bacteroidia
c_Spirochaetia
c_Clostridia
c_Methanomicrobia *
o_Campylobacterales
o_Desulfovibrionales
o_Bacteroidales
o_Burkholderiales
o_Spirochaetales
o_Eubacteriales
o_Methanomicrobiales *
f_Helicobacteraceae
f_Desulfovibrionaceae
f_Bacteroidaceae *
f_Prevotellaceae
f_Burkholderiaceae
f_Rikenellaceae
f_Tannerellaceae
f_Barnesiellaceae
f_Paludibacteraceae
f_Treponemataceae
f_Sphaerochaetaceae
f_Oscillospiraceae
f_Clostridiaceae
f_Lachnospiraceae
f_Methanocorpusculaceae *
g_Helicobacter
g_Desulfovibrio
g_Bacteroides *
g_Prevotella
g_Limnobacter
g_Alistipes
g_Parabacteroides *
g_Barnesiella
g_Treponema
g_Paludibacter
g_Sphaerochaeta *
g_Ruminococcus
g_Clostridium
g_Coprococcus
g_Methanocorpusculum *
acetate---CoA ligase [6.2.1.1]
aceticacid (C00033)
Acetyl adenylate (C05993)

(K)

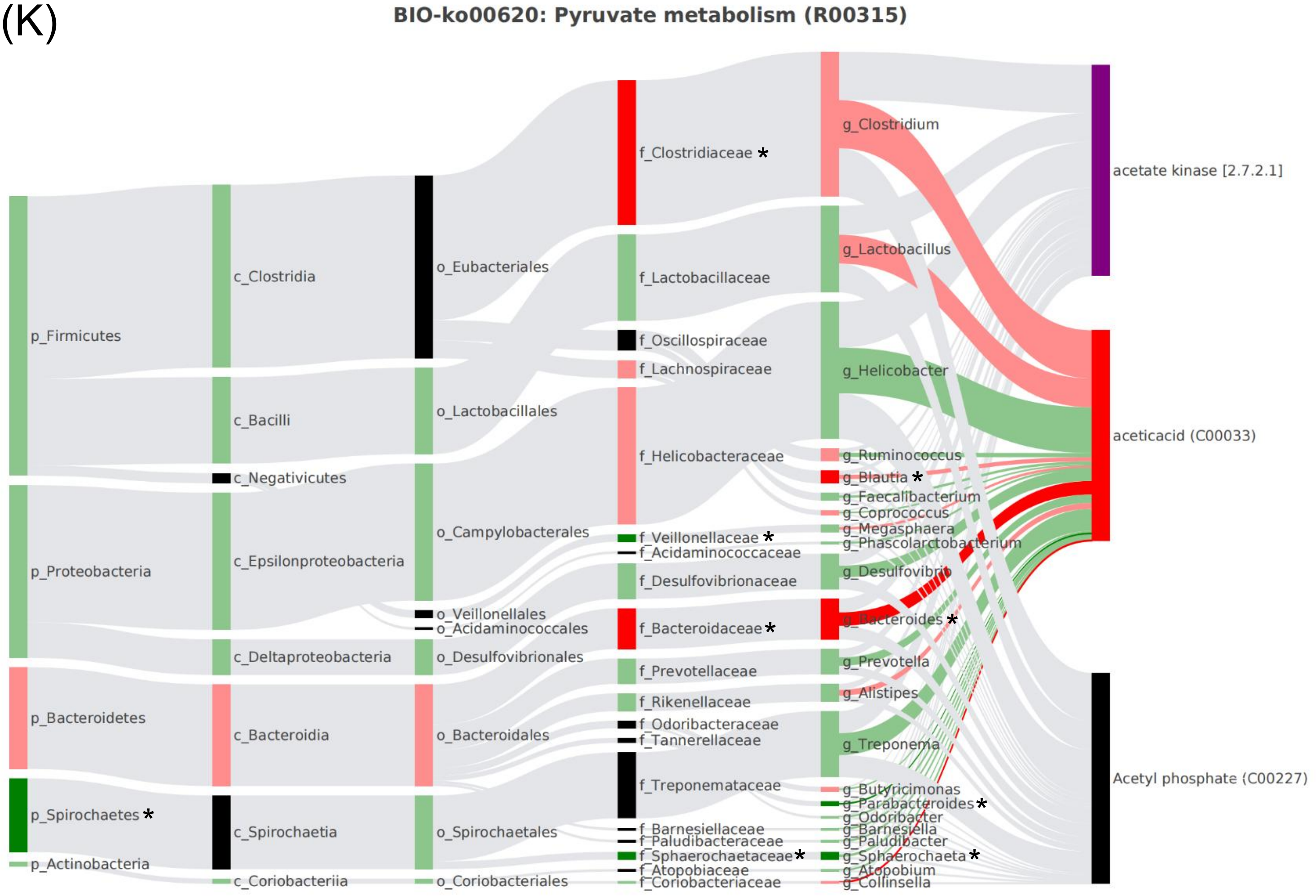
BIO-ko00620: Pyruvate metabolism (R00315)
p_Firmicutes
p_Proteobacteria
p_Bacteroidetes
p_Spirochaetes *
p_Actinobacteria
c_Clostridia
c_Bacilli
c_Negativicutes
c_Epsilonproteobacteria
c_Deltaproteobacteria
c_Bacteroidia
c_Spirochaetia
c_Coriobacteriia
o_Eubacteriales
o_Lactobacillales
o_Campylobacterales
o_Veillonellales
o_Acidaminococcales
o_Desulfovibrionales
o_Bacteroidales
o_Spirochaetales
o_Coriobacteriales
f_Clostridiaceae *
f_Lactobacillaceae
f_Oscillospiraceae
f_Lachnospiraceae
f_Helicobacteraceae
f_Veillonellaceae *
f_Acidaminococcaceae
f_Desulfovibrionaceae
f_Bacteroidaceae *
f_Prevotellaceae
f_Rikenellaceae
f_Odoribacteraceae
f_Tannerellaceae
f_Treponemataceae
f_Barnesiellaceae
f_Paludibacteraceae
f_Sphaerochaetaceae *
f_Atopobiaceae
f_Coriobacteriaceae
g_Clostridium
g_Lactobacillus
g_Helicobacter
g_Ruminococcus
g_Blautia *
g_Faecalibacterium
g_Coprococcus
g_Megasphaera
g_Phascolarctobacterium
g_Desulfovibrio
g_Bacteroides *
g_Prevotella
g_Alistipes
g_Treponema
g_Butyricimonas
g_Parabacteroides *
g_Odoribacter
g_Barnesiella
g_Paludibacter
g_Sphaerochaeta *
g_Atopobium
g_Collinsella
acetate kinase [2.7.2.1]
aceticacid (C00033)
Acetyl phosphate (C00227)

(L)

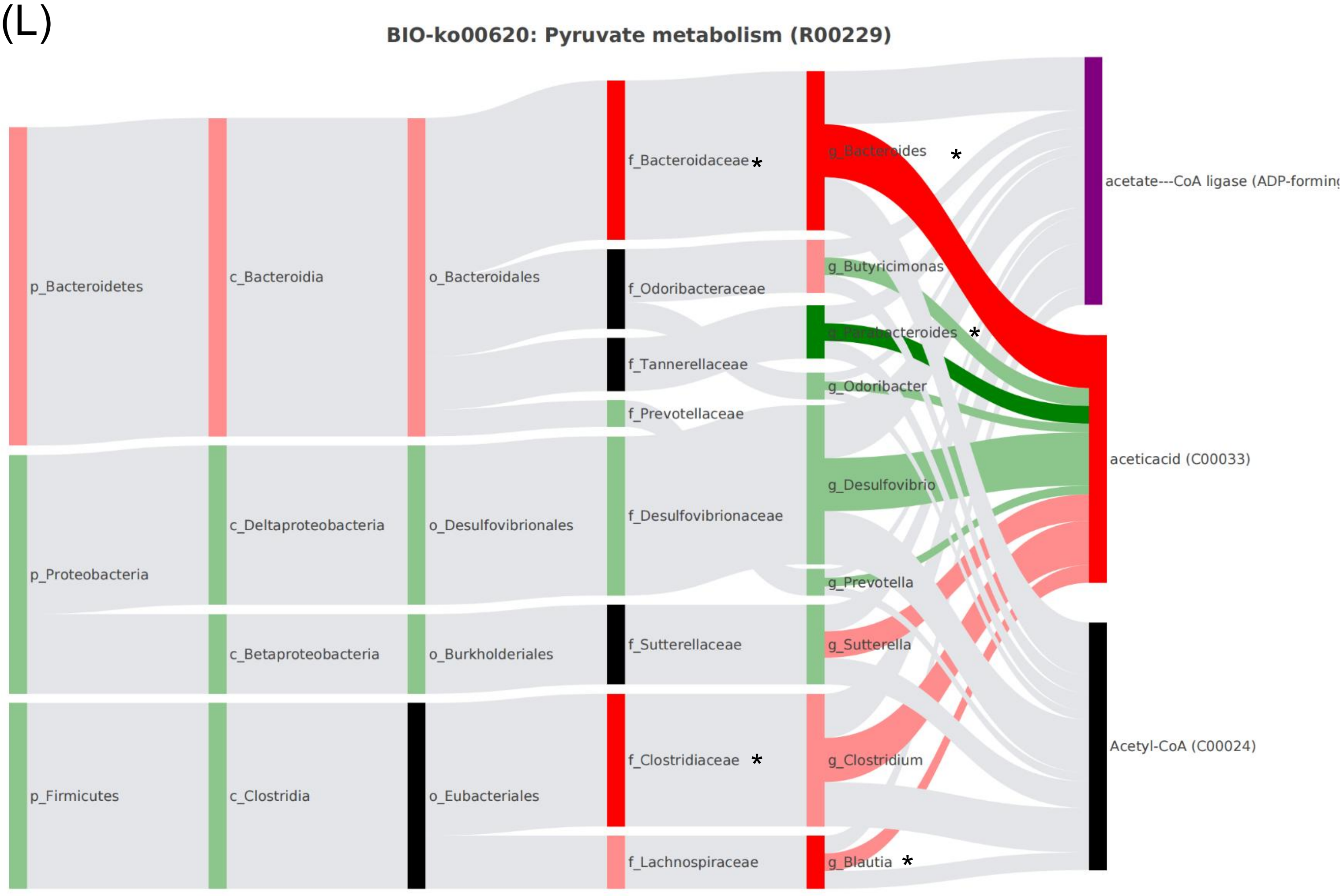
BIO-ko00620: Pyruvate metabolism (R00229)
p_Bacteroidetes
p_Proteobacteria
p_Firmicutes
c_Bacteroidia
c_Deltaproteobacteria
c_Betaproteobacteria
c_Clostridia
o_Bacteroidales
o_Desulfovibrionales
o_Burkholderiales
o_Eubacteriales
f_Bacteroidaceae *
f_Odoribacteraceae
f_Tannerellaceae
f_Prevotellaceae
f_Desulfovibrionaceae
f_Sutterellaceae
f_Clostridiaceae *
f_Lachnospiraceae
g_Bacteroides *
g_Butyricimonas
g_Parabacteroides *
g_Odoribacter
g_Desulfovibrio
g_Prevotella
g_Sutterella
g_Clostridium
g_Blautia *
acetate---CoA ligase (ADP-forming
aceticacid (C00033)
Acetyl-CoA (C00024)

(M)

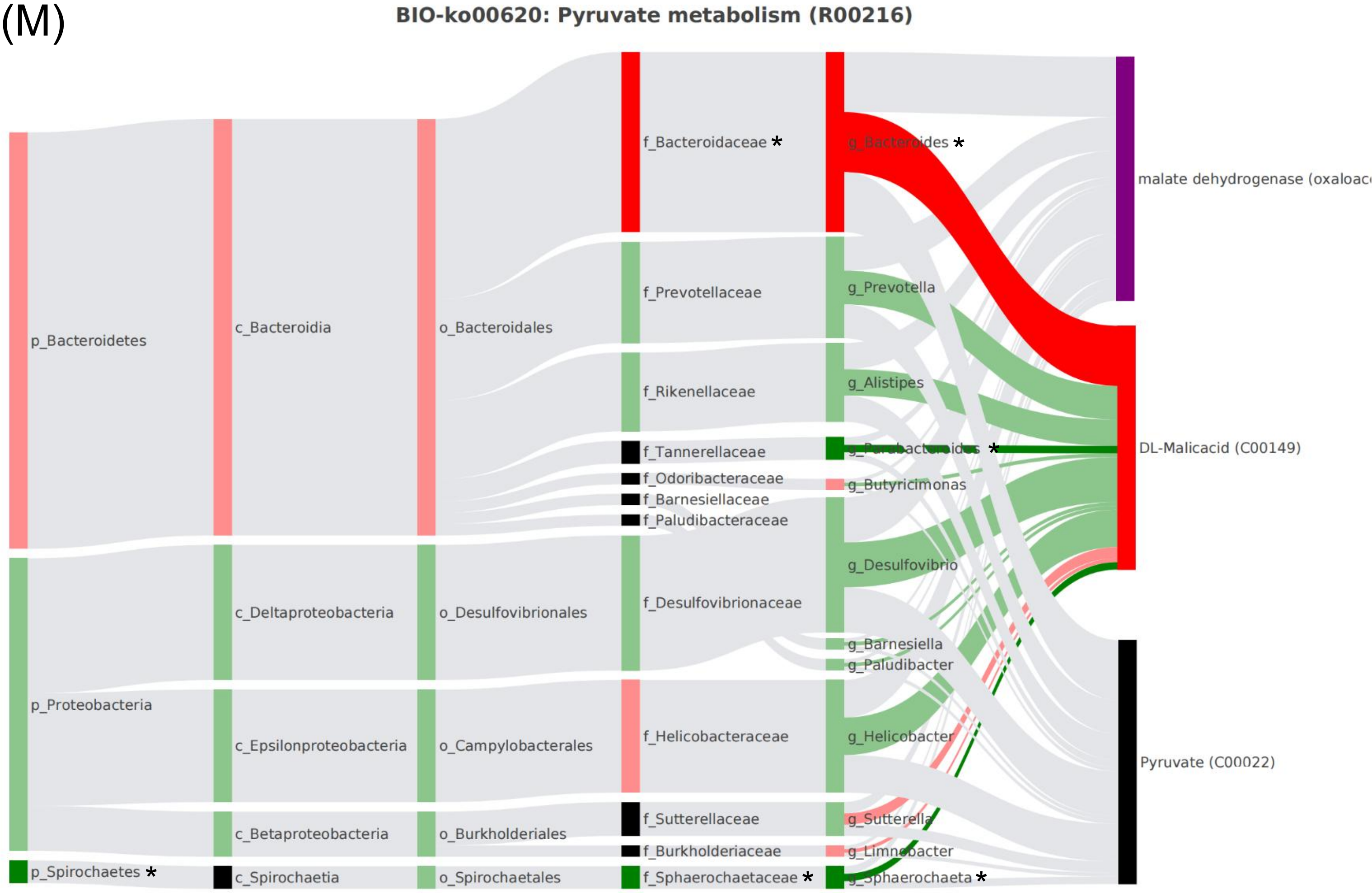
BIO-ko00620: Pyruvate metabolism (R00216)
p_Bacteroidetes
p_Proteobacteria
p_Spirochaetes *
c_Bacteroidia
c_Deltaproteobacteria
c_Epsilonproteobacteria
c_Betaproteobacteria
c_Spirochaetia
o_Bacteroidales
o_Desulfovibrionales
o_Campylobacterales
o_Burkholderiales
o_Spirochaetales
f_Bacteroidaceae *
f_Prevotellaceae
f_Rikenellaceae
f_Tannerellaceae
f_Odoribacteraceae
f_Barnesiellaceae
f_Paludibacteraceae
f_Desulfovibrionaceae
f_Helicobacteraceae
f_Sutterellaceae
f_Burkholderiaceae
f_Sphaerochaetaceae *
g_Bacteroides *
g_Prevotella
g_Alistipes
g_Parabacteroides *
g_Butyricimonas
g_Desulfovibrio
g_Barnesiella
g_Paludibacter
g_Helicobacter
g_Sutterella
g_Limnobacter
g_Sphaerochaeta *
malate dehydrogenase (oxaloac
DL-Malicacid (C00149)
Pyruvate (C00022)

(N)

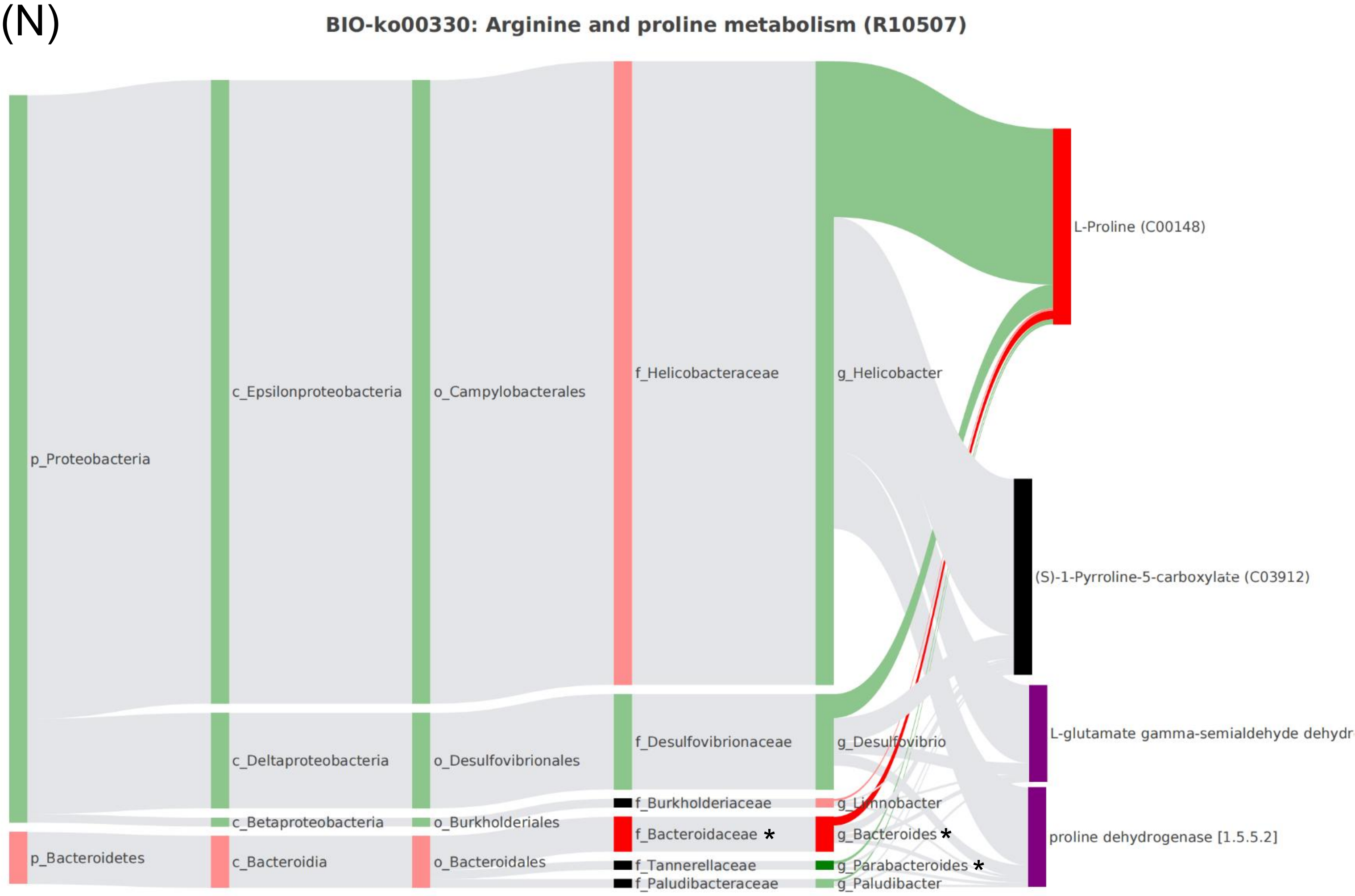
BIO-ko00330: Arginine and proline metabolism (R10507)
p_Proteobacteria
p_Bacteroidetes
c_Epsilonproteobacteria
c_Deltaproteobacteria
c_Betaproteobacteria
c_Bacteroidia
o_Campylobacterales
o_Desulfovibrionales
o_Burkholderiales
o_Bacteroidales
f_Helicobacteraceae
f_Desulfovibrionaceae
f_Burkholderiaceae
f_Bacteroidaceae *
f_Tannerellaceae
f_Paludibacteraceae
g_Helicobacter
g_Desulfovibrio
g_Limnobacter
g_Bacteroides *
g_Parabacteroides *
g_Paludibacter
L-Proline (C00148)
(S)-1-Pyrroline-5-carboxylate (C03912)
L-glutamate gamma-semialdehyde dehydr
proline dehydrogenase [1.5.5.2]

(O)

BIO-ko00330: Arginine and proline metabolism (R01251)

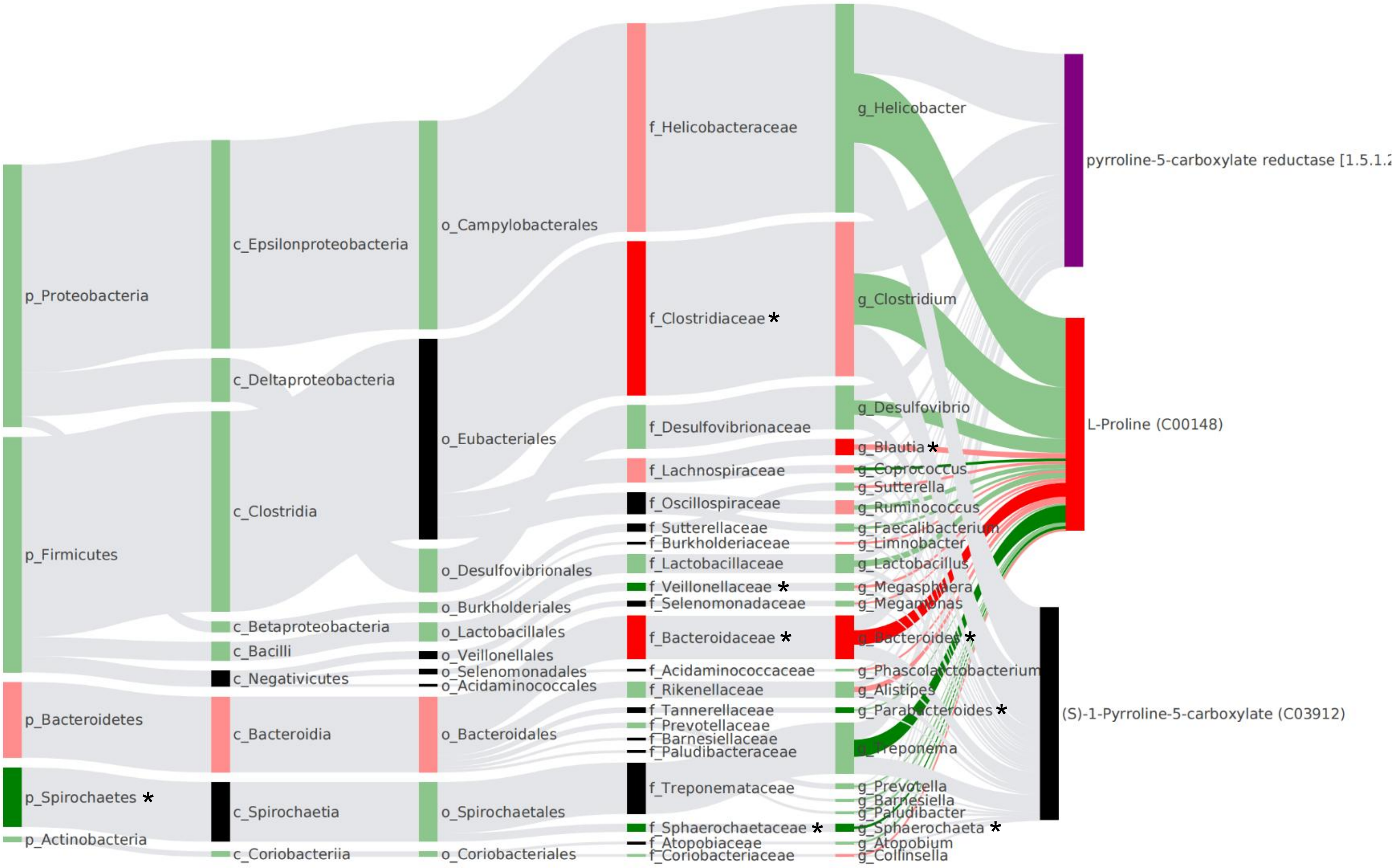

(P)

BIO-ko00330: Arginine and proline metabolism (R01248)

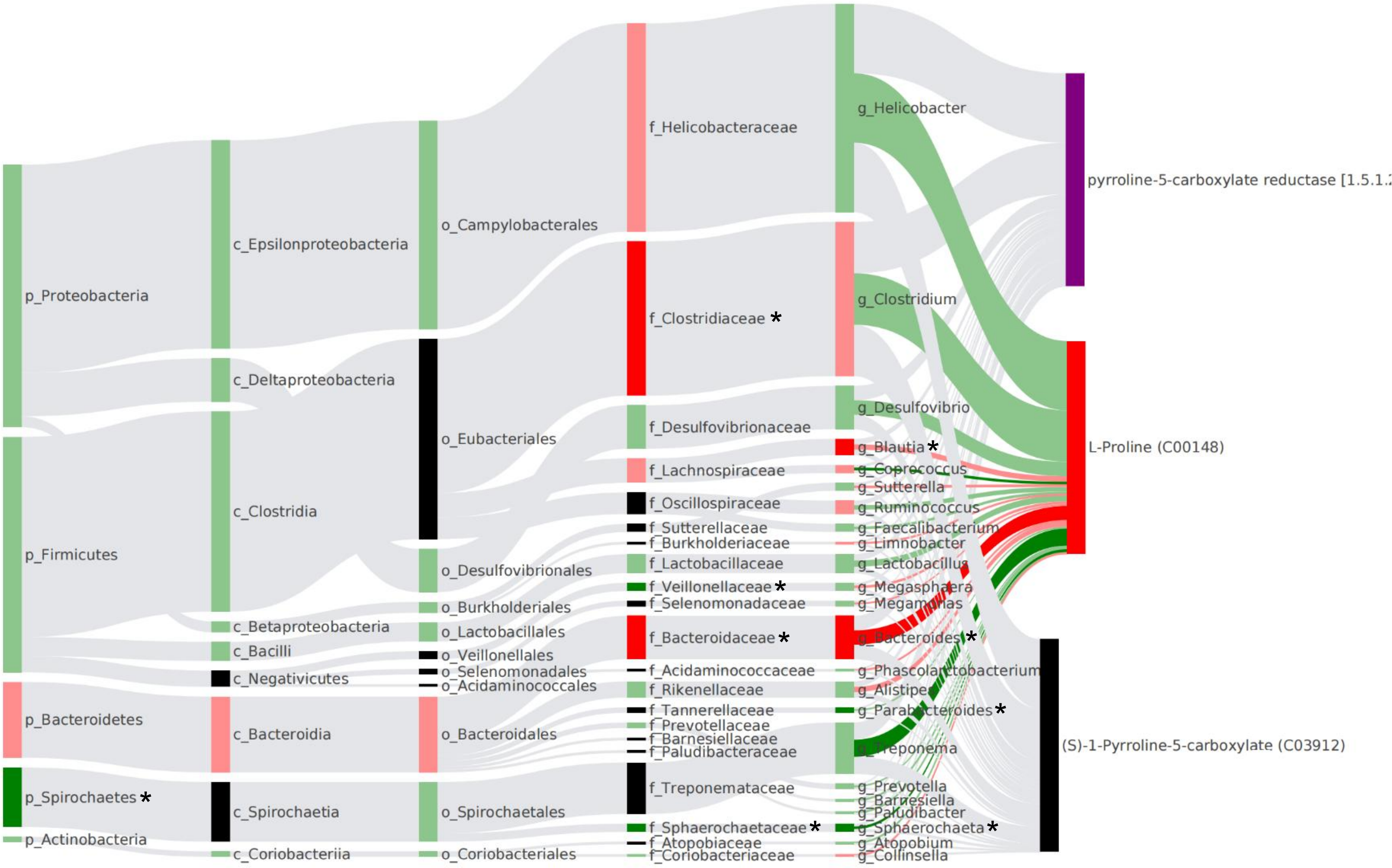

(Q)

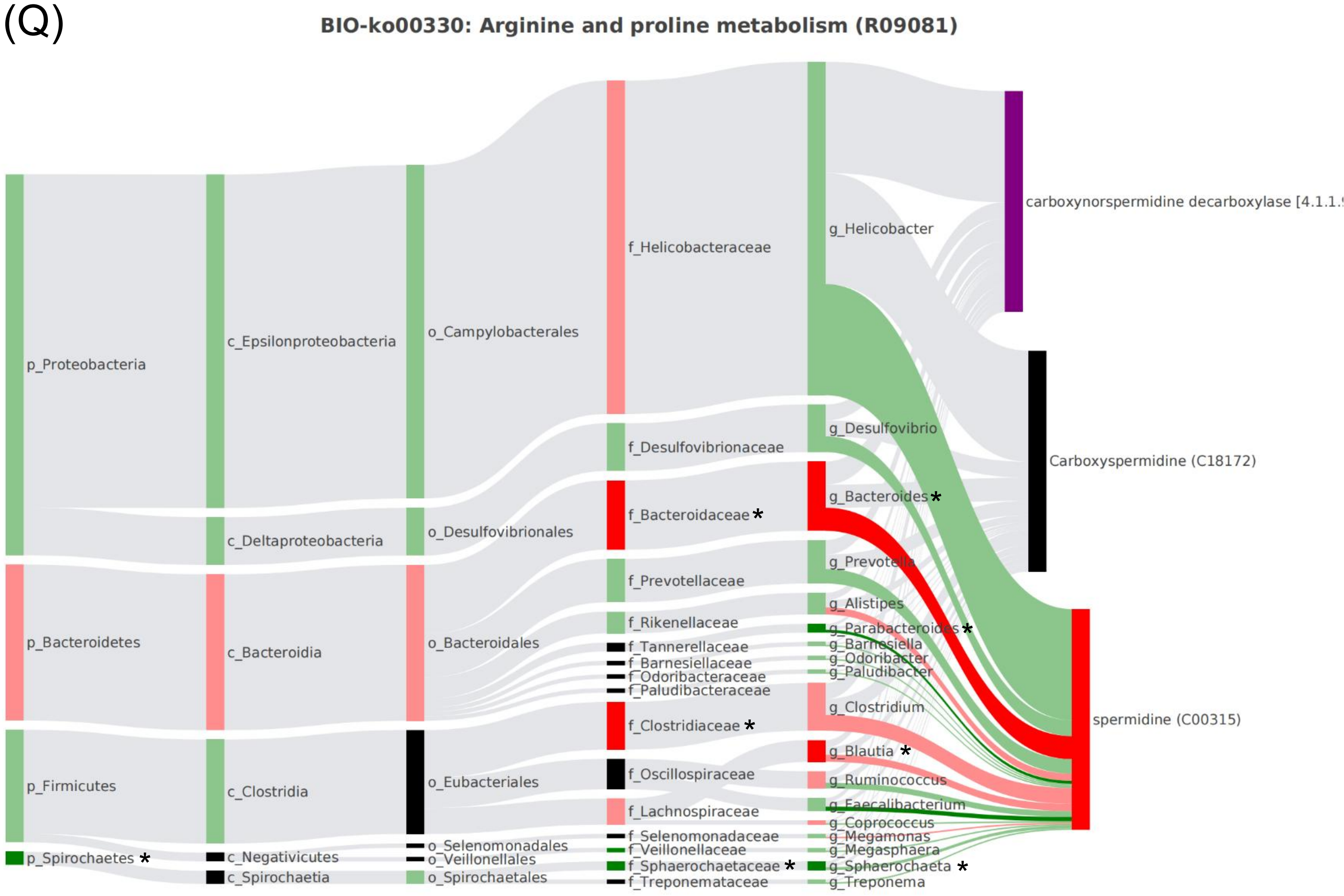

Supplementary Figure S5 The BIO-Sankey Network for several metabolic reaction in significantly different metabolism pathways.

The BIO-Sankey Network for (A) R00235 and (B) R00219 metabolic reaction in glycolysis / gluconeogenesis metabolism.

The BIO-Sankey Network for (C) R10705, (D) R01354, (E) R01353 and (F) R00920 metabolic reaction in propanoate metabolism.

The BIO-Sankey Network for (G) R03145, (H) R01082, (I) R00342, (J) R00316, (K) R00315, (L) R00229 and (M) R00216 metabolic reaction in pyruvate metabolism.

The BIO-Sankey Network for (N) R10507, (O) R01251, (P) R01248 and (Q) R09081 metabolic reaction in arginine and proline metabolism.

Note: Asterisks (*) indicate statistically significant correlations with metabolites. The red/green color of nodes indicates up/downregulation. The red/green bands indicate the positive/negative correlations with metabolites. The dark red/green color indicates the statistically significance $p < 0.05$.
